# Nanoparticles: The Plant Saviour under Abiotic Stresses

**DOI:** 10.3390/nano12213915

**Published:** 2022-11-06

**Authors:** Muhammad Fasih Khalid, Rashid Iqbal Khan, Muhammad Zaid Jawaid, Waqar Shafqat, Sajjad Hussain, Talaat Ahmed, Muhammad Rizwan, Sezai Ercisli, Oana Lelia Pop, Romina Alina Marc

**Affiliations:** 1 Environmental Science Centre, Qatar University, Doha 2713, Qatar; 2 Southwest Florida Research and Education Center, Horticultural Sciences Department, Institute of Food and Agricultural Science, University of Florida, Immokalee, FL 34142, USA; 3 Institute of Horticultural Sciences, University of Agriculture Faisalabad, Faisalabad 38040, Pakistan; 4 Department of Forestry, College of Forest Resources, Mississippi State University, Starkville, MI 39759, USA; 5 Department of Horticulture, Faculty of Agricultural Science & Technology, Bahauddin Zakariya University, Multan 60800, Pakistan; 6 Office of Academic Research, Office of VP for Research and Graduate Studies, Qatar University, Doha 2713, Qatar; 7 Department of Horticulture, Faculty of Agriculture, Ataturk University, 25240 Erzurum, Turkey; 8 Department of Food Science, Faculty of Food Science and Technology, University of Agricultural Science and Veterinary Medicine, 400372 Cluj-Napoca, Romania; 9 Department of Food Engineering, Faculty of Food Science and Technology, University of Agricultural Sciences and Veterinary Medicine, 400372 Cluj-Napoca, Romania

**Keywords:** nanoparticles, stress tolerance, physiology, molecular, drought, salinity, temperature, heavy metals, nutrients imbalance

## Abstract

Climate change significantly affects plant growth and productivity by causing different biotic and abiotic stresses to plants. Among the different abiotic stresses, at the top of the list are salinity, drought, temperature extremes, heavy metals and nutrient imbalances, which contribute to large yield losses of crops in various parts of the world, thereby leading to food insecurity issues. In the quest to improve plants’ abiotic stress tolerance, many promising techniques are being investigated. These include the use of nanoparticles, which have been shown to have a positive effect on plant performance under stress conditions. Nanoparticles can be used to deliver nutrients to plants, overcome plant diseases and pathogens, and sense and monitor trace elements that are present in soil by absorbing their signals. A better understanding of the mechanisms of nanoparticles that assist plants to cope with abiotic stresses will help towards the development of more long-term strategies against these stresses. However, the intensity of the challenge also warrants more immediate approaches to mitigate these stresses and enhance crop production in the short term. Therefore, this review provides an update of the responses (physiological, biochemical and molecular) of plants affected by nanoparticles under abiotic stress, and potentially effective strategies to enhance production. Taking into consideration all aspects, this review is intended to help researchers from different fields, such as plant science and nanoscience, to better understand possible innovative approaches to deal with abiotic stresses in agriculture.

## 1. Introduction

A variety of factors influence agricultural productivity, including the climate. Agriculture is fundamental to human welfare, and many organizations and others are concerned about the effects of climate change on agriculture. As a result of increasing annual temperatures, changing patterns of rainfall, floods, and dwindling water reserves, major agriculture crops are affected by climate change. The agricultural sector provides income and employment to almost half of the labor force and supplies raw materials to industry in developing and less developed countries. Global hunger and food insecurity are continuously increasing due to the phenomenal increase in global population and stagnant agricultural performance [1]. Climate change causes many biotic and abiotic stresses to plants which affect plant growth and cause declines in yield [2]. Different strategies have been adopted to overcome these negative effects of climate change, i.e., the use of tolerant genotypes, application of different plant growth regulators, and the use of organic fertilizers. Currently, nanotechnology is substantially contributing to this sector. Nanotechnology studies the various structures of matter on the scale of a billionth of a meter. A nanoparticle (NP) is a small molecular aggregate with an interfacial layer surrounding a diameter of 1 to 100 nanometers. Several critical properties of matter are fundamentally impacted by this interfacial layer at the nanoscale [3,4]. As a result of their small size, NPs have some unusual properties compared with bulk materials. Nanoparticles refer to organic materials rather than individual molecules. The fact that NPs link bulk materials to atomic or molecular structures cause them to be of high scientific interest. The various NPs used for the treatment of plants to overcome environmental challenges are: titanium dioxide (TiO_2_), zinc (Zn), zinc oxide (ZnO), cesium (Ce), cobalt (Co), copper (Cu), copper oxide (CuO), selenium (Se) NPs, silver (Ag), silicon (Si), silicon oxide (SiO_2_), iron oxide (FeO), calcium (CaCO_3_), magnesium (Mg), magnesium oxide (MgO), manganese (Mn), and molybdenum (Mo) NPs; and aluminium oxide (AlO_4_) and carbon nanotubes (CNTs).

**Figure 1:**
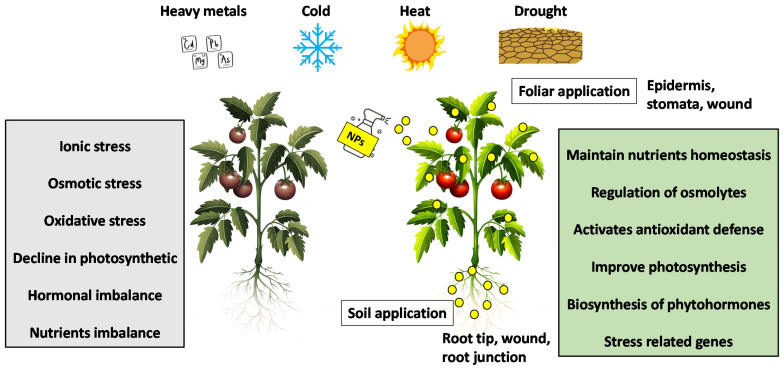
Mechanisms of NPs mitigate abiotic stresses in plants.

To cope with environmental stress, plants have developed a wide range of efficient and comprehensive molecular programs to rapidly sense stressors and adapt accordingly [5]. Plants can enhance this response through the interaction of NPs with plants. Nanotechnology promises to increase crop yield by improving plant tolerance mechanisms under abiotic stress conditions [6]. Several studies have shown that NPs play a vital role in improving the tolerance of plants to abiotic stresses by modulating various physiological, biochemical, and molecular processes (Figure 1).

Crop growth and improvement can effectively be achieved in modern agriculture through nanotechnology. NPs can be used in the agricultural sector as nano-agrochemicals (nanobiocomposites, nanopesticides, nanofertilizers), agri-food production, nanobiosensors, agri-environment, organic agriculture, postharvest management, and plant genetic progress by NP-mediated gene transfer [7,8]. In recent years, the reliance on nanotechnology in different industries has been increasing due to its copious potential, sustainable, eco-friendly, and cost-effective applications. The use of nanopesticides and nanofertilizers has enhanced agricultural productivity, for example, urea-doped calcium phosphate nanofertilizers have helped commercial crops to obtain efficient nutrients from the soil, specifically urea; helped maintain crop growth and productivity; and helped to achieve sustainable agriculture [9,10,11]. Madusanka et al. [12] observed the slow release of nitrogen by using a urea-hydroxyapatite-montmorillonite nanohybrid composite. The use of hydroxyapatite nanoparticles significantly influenced the crop yield and germination attributes of tomato plants [13]. The range of applications of nanotechnology in the remediation of soil and water has increased food quality and production. Moreover, with nanotechnology being eco-friendly, its use has a significant benefit in reducing the harmful effects of chemicals used on crops, and the effects caused by agriculture on the environment [14]. NPs have been effective on seed and plant metabolisms by enhancing growth. The advantageous characteristics of NPs being small allows them to cross biological barriers in plants more efficiently and remediate plant stresses, such as salt stress and heat stress, and stress caused by heavy metals [15]. 

NPs and their effects on plants under abiotic stress conditions are well documented. However, to date, no proper review has summarized and explained the physiological, biochemical, and molecular mechanisms of plants under abiotic stresses and their coping mechanism by use of nanotechnology. This review is designed to revitalize the status of NP and plant research, and identify the key knowledge gaps in order to tackle the mountainous challenge of abiotic stresses caused by climate change, in the agricultural sector. Our goal is to accumulate and integrate previous research to provide relevant information on NPs and plant abiotic stresses. Academia and researchers interested in nanotechnology, biology, plants, abiotic stress physiology, or biotechnology will find this article of interest. This new body of knowledge can be used to assess and minimize abiotic stresses in plants with the help of nanotechnology.

## 2. Review Scope and Approach

**Figure 2:**
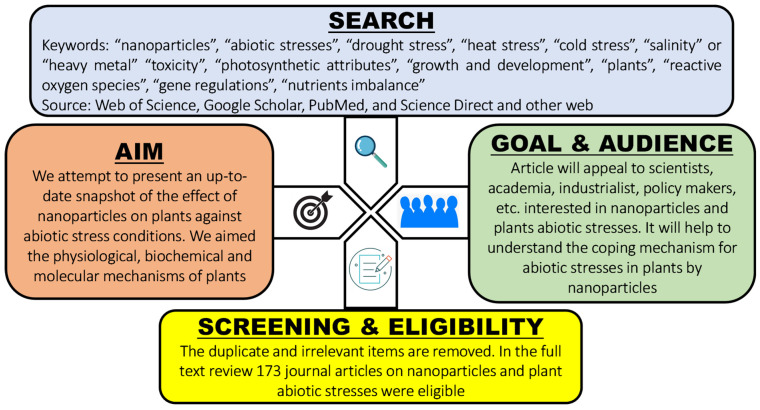
Schematic diagram depicting the decision-making process for the selection of a journal article, and the scope of the review.

The current review study extensively covers MEDLINE and other published literature between 2015 and 2022 (until July), reporting the effect of NPs on plant growth and physiology under different abiotic stresses caused by climate change. The impact of nanoparticles has recently been documented in novel ways. In this review, each scientific article was critically reviewed for its method, result, and conclusion when discussing specific NPs. The search was performed in the databases “Google Scholar”, “PubMed”, and “Web of Science”, using different keywords. Consequently, this review summarizes and consolidates the current research findings about NPs in the following areas: (1) response of plants to abiotic stresses and their mitigation strategies using NPs; (2) the physiological and growth attributes of major abiotic stresses, i.e., drought, temperature extremities, salinity, and heavy metals; and (3) the biochemical and molecular responses of plants when exposed to NPs under abiotic stress conditions. We provide a detailed assessment of the effect of NPs on plant mechanisms under abiotic stresses. However, despite our efforts, we were unable to cover every aspect thoroughly. Concisely, the review approach was as follows. The keywords “NPs”, “abiotic stresses”, “drought stress”, “heat stress”, “cold stress”, “salinity” or “heavy metal” “toxicity”, “photosynthetic attributes”, “growth and development”, “plants”, “reactive oxygen species”, and “gene regulations” were selected individually or jointly. Scientific literature, preferably spanning between 2015 and 2022 (until July), was assimilated and reviewed. Figure 2 shows a schematic diagram depicting the decision-making process for the selection of appropriate journal articles and the scope of the review. Each selected article was then explained in terms of its key concepts.

## 3. Drought Stress

Drought is a chronic abiotic stress affecting crop growth and development, accounting for approximately 70% of the potential loss of global crop yield and productivity [16]. Drought hinders agriculture and forestry worldwide, due to very little rainfall or significant differences in moisture. The current trends of global warming are causing a major impact on the moisture levels of the soil and the environment, and are increasing the intensity of droughts. Plants are subjected to various stresses during their growth, and the morphology of plants is affected at all stages of development due to drought stress, with productivity losses expected to reach 30% globally by 2025. Severe droughts are a major problem for agriculture in a changing climate, as water scarcity is predicted to become more common. Drought refers to the conditions where a plant’s water demand cannot be fully met, such as where the transpiration water level of the plant exceeds the water absorbed by the root system, insufficient precipitation, a drop in the groundwater level, or water retention by soil particles [17]. Plants reduce water loss through adjustments in morphological anatomy, physiology and biochemistry to maintain their water status as a result of drought [18,19]. Drought stress leads to a reduction in cell enlargement as compared with affecting cell division. It affects plant growth by altering the functioning of various physiological and biochemical processes, i.e., photosynthesis, respiration, enzymatic activity, and nutrient metabolism [20]. The response of plants to drought stress varies at different tissue levels, depending on the intensity and duration of the stress, as well as plant species and growth stage. Understanding how plants respond to drought is very important and an essential part of improving the tolerance of crops to stress.

Different molecular, biochemical, physiological, morphological and ecological traits and processes are disrupted under drought stress conditions [21,22]. A deficit of water has adverse effects on plant yield and quality. Growth stage, age, plant species, drought severity, and duration are key factors affecting plant response to drought [23]. Plants die off under prolonged drought conditions [24]. Water scarcity in plants increases the concentration of the solute in the cytosol and extracellular matrices as a result of the reduction in plant cells’ water potential and turgor, which leads to growth inhibition and reproductive failure. Wilting is caused by the accumulation of abscisic acid and compatible osmolytes [25,26]. Adverse influences are aggravated due to the overproduction of ROS and radical scavenging compounds such as ascorbate and glutathione [27,28]. Water stress in plants due to drought affects the stomatal functions and limits the gaseous exchange, decreasing the rate of transpiration and carbon assimilation [29]. In turn, the mechanisms of resistance of plants to drought vary. Therefore, plants can reduce resource utilization and regulate growth in response to adverse environmental conditions [30]. Signal transduction, a network at the molecular level, enhances these responses to drought stress [31]. Plant stomatal regulation by enhancing ion transport, transcription factor activity, and ABA signal transduction is also involved in the molecular mechanism of plant response [32]. In some changing environments, there is a need to enhance the resistance of plants against drought. To improve water use efficiency when the physical fitness of roots and leaves is insufficient to cope with certain drought molecular signals, plant enhancement may be conducted by including genes encoding regular proteins and signals by crosstalk, expressing many other genes according to different regulatory mechanisms [33]. To achieve future food demands, further advancement is required in enhancing drought tolerance in plants, and the adoption of economical and beneficial agricultural practices will be critical [34].

### 3.1. NPs Mitigate Drought Stress in Plants

NPs are known by their specific shape, tunable pore size, and high reactivity with enhanced surface area [35]. NPs are considered an effective and promising tool for regulating crop yield and overcoming current and future limitations of agricultural production by increasing the tolerance mechanisms in plants under abiotic stress conditions. The mitigating effect of NPs on drought stress is caused by inducing physiological and biochemical regulation, and regulating the expression of genes relating to drought response/tolerance. NPs enhance the photosynthetic activity of drought-induced plants, whereby the improvement of root growth, upregulation of aquaporins, altered intracellular water metabolism, accumulation of compatible solutes, and ionic homeostasis are the main mechanisms by which NPs alleviate osmotic stress caused by water deficiency. NPs reduce leaf water loss caused by the accumulation of ABA through stomatal closure, and ameliorate oxidative stress damage by reducing reactive oxygen species and activating antioxidant defense systems.

#### 3.1.1. Physiological and Biochemical Aspect

Nanotechnology has the capability to enhance plant photosynthesis efficiency by altering the enzymatic activity involved in the C3 cycle, along with regulating photosynthetic pigments responsible for plant growth [36]. NPs have positive effects on plant germination and growth, however, their efficacy varies with their concentration and host plant. In sorghum plants under drought conditions, foliar spraying of nanowax increased seed yield in plants in comparison with spraying with water. TiO_2_ NPs have many strong effects on the morphological, biochemical, and physiological properties of crops [37]. During the growth phase of cucumber plants, exogenous application of NPs promoted rubisco activase activity, chlorophyll formation, and photosynthetic rate, which led to an increase in plant dry mass [38]. It was further noted that foliar application of NPs could increase the seed yield of soybean, due to enhanced photosynthesis [39].

The impact of nano-TiO_2_ varies with respect to changing environmental conditions, plant species, and different application doses. In this context, Mohammadi et al. [40] investigated the effects of nano-TiO_2_ concentrations on the biochemical and morphophysiological properties of dragonhead plants. The TiO_2_ increases the growth and essential oil in plants under water deficit stress. A formulation of nano-sized ZnO and CuO was used as a fertilizer. The results showed that at different NP doses, root growth was reduced, while contrarily, at other levels, Zn NPs expanded lateral root formation whereas Cu NPs induced proliferation and elongation of root hairs close to the roots of wheat seedlings under simulated drought stress [41]. These responses typically occur when the roots are colonized by a beneficial bacteria isolated from wheat roots grown in calcareous soils under dryland farming conditions.

It has been observed that ZnO and CuO NPs exhibited protection against drought stress in different plants [41]. This protection may be induced by the enhanced generation of lateral root hairs which resulted in proper water absorption. Enhanced cell wall lignification in mustard and Arabidopsis under CuO may alter water flow, thereby limiting cell wall elongation. The response of plants to drought stress is an increase in lignification. The disruption of water flow occurs due to the binding of copper ions to the pectin of the cell wall [42]. Some notable results were found in some studies, such as increased seed germination and antioxidant content after barley, soybean, and maize were treated with carbon nanotubes (CNTs) [43]. CNTs can induce root and shoot growth in wheat plants. Various major efforts have been conducted over the past few decades to reduce the effects of drought stress on plant quality and productivity. We further suggest that fullerenol (FNPs) NPs with molecular formula C60(OH)24 may help alleviate the effects of drought stress and provide additional water supply between plant cells. Precisely, nanofullerenols (FNPs) can enter the root and leaf tissues of plants, where they can bind water molecules in various parts of the cell. This water absorbing FNP activity further suggests that FNPs may be useful for plants [44,45]. The results of this study by Borišev et al. [44] further demonstrated that foliar application of nanofullerenol could alter intracellular water metabolism in drought-stressed plants. Under drought stress, the content of the permeate product proline in plant roots and leaves was significantly increased. These results further suggest that FNPs could also function as a binder for intracellular water, thereby generating additional water reserves, and allowing them to adapt to drought stress. Ag NPs are the most used NPs in research experiments [46]. 

In plants, NPs target the cellular organelles and release various contents [8], thus modulating the activity of antioxidants enzymes, i.e., SOD, CAT, and POD [47]. This effect was exhibited by incremented SOD activity in plants under TiO_2_ NP application [48]. In agriculture, certain elements, along with oxides as NPs, have been used for incremental resistance against drought stress. Si NPs have been used extensively for ameliorating the negative impacts of various abiotic stresses including drought [49]. The improvement in growth, physio- and biochemical characteristics has been observed upon treatment with silica and ZnO NPs on different crops [35]. Similarly, Si NPs ameliorated drought stress on wheat plants [50]. Similarly, ZnO NPs reduced the negative impact of salinity and drought stress on plants [51]. It has been observed that excessive NP application led to a generation of oxidative stress, i.e., leading to cell cycle arrest, programmed cell death, protein regulation, and induction of antioxidant enzymes [52], whereas NP-treated plants exhibited significant reductions in MDA levels along with free radicals, i.e., H_2_O_2_ and O_2^−^_, under drought conditions. However, it was also observed that TiO_2_ application enhanced antioxidant enzyme activities, i.e., POD and CAT, whereas MDA levels were reduced due to the induction of the plant’s antioxidant system [27]. 

Under drought stress, the level of anthocyanin in plants exposed to CuO NPs continued to increase, and the level of proline was also shown to increase under drought stress. Wheat roots treated with CuO-treated NPs exhibited a greater accumulation of free radicals, consistent with plants responding to the challenge of NP-induced ROS bursts. Elevated ROS levels, further suggesting that drought stress triggers a consequence of elevated ABA, may lead to transcriptional changes that lead to tolerance. The amplification of various antioxidant enzymes (GR, SOD, GPX, APX, and CAT) in plants suggested that foliar application of fullerenol (FNPs) NPs with molecular formula C60(OH)24 might have some valuable effects on mitigating the oxidative effect of drought stress, which further depends on the concentration of NPs applied [43]. The exact mode of action and physiological mechanism of FNPs on plants needs to be further studied.

#### 3.1.2. Molecular Aspect

Transcriptomic and proteomic approaches have deeply investigated the effects of NPs on different plant species at the molecular level. Morphological and physiological effects have been reported to largely depend on the dose used, as well as the type, size and shape of NPs [53,54]. Expression of the *P5CS* gene leads to increased plant tolerance to different environmental stress conditions, including biotic and abiotic stresses, since this gene encodes proline biosynthesis. *MAPK2*, a member of the *MAP* kinase gene family, plays an extremely important role in regulating phytohormones and antioxidant protection mechanisms in response to different stress environments [54] in combination with *Ca21* and ROS. *AREB/ABF* are transcriptional regulators necessary for the regulation of the *AREB* gene encoding abscisic acid, and are critical in stimulating resistance to stressful environments such as drought and salt stress [54,55]. Downregulation of the *ZFHD* gene reduces the negative effects of salt and drought stress and is controlled by the abscisic acid biosynthesis pathway. On the other hand, downregulation of the *TAS14* gene reduces osmotic pressure and enhances solute aggregation, including K1 and sugars, making plant species more resistant to drought and salt stress [54]. Application of Ag NPs (5 and 10 mg/L) to rape plants modulated the metabolic pathways of glucosinolate and phenolic related genes, which are also associated with biotic and abiotic stresses, and inhibited carotenoid genes [56]. Downregulation of the *ZFHD* gene reduces the negative effects of salt and drought stress and is controlled by the abscisic acid biosynthesis pathway. The use of Ag and Ag1 NPs on Arabidopsis plants resulted in overexpression of oxidative stress and metal response-related genes, and downregulation of ethylene and auxin-related genes [54]. Three of these genes overexpressed by Ag NPs are involved in the biosynthesis of thalianol, which is thought to contribute to a plant’s antioxidant protection mechanism. The response of different NPs against drought stress conditions is summarized in Table 1.

## 4. Temperature

Since the turn of the century, the earth’s average temperature has risen [63]. Global warming can adversely affect the environment because of the increase in the temperature of the earth. In climates where temperatures exceed the ideal range, crops begin to yield less. Extreme climate change can cause high temperatures and drought, causing severe damage to agriculture and posing a threat to tree populations [64]. NPs are sourced from heavy metals which can cause environmental degradation by their toxic effects on soil, water, and plant resources [65,66]. The primary translocation of NPs from soil to plant body occurs through lateral roots. The NPs travel within xylem tissues and reach the whole plant body [52]. The translocation depends upon the size of the NPs. The contrastingly positive effect of NPs in mitigating abiotic stress has also been reported. Different NPs for field applications such as nano-agrochemicals have been used to increase agricultural productivity. Temperature extremes negatively affect plants’ physiological and molecular mechanisms [63,67].

### 4.1. NPs Mitigate Temperature Extremities in Plants

NPs play a pivotal role in plants under stress, which could help them tolerate abiotic stresses, especially temperature stress [68,69]. Plant growth and hydration were increased when nanoparticles were applied in different concentrations to reduce the effects of heat stress [70]. Plants exhibit antioxidative properties when NPs are applied at low concentrations, but suffer from oxidative stress when NPs are at high concentrations. Molecular chaperones and heat shock proteins are synthesized by plants under heat stress. A heat shock protein assists other proteins in maintaining their stability in stressful conditions, as well as being involved in heat stress resistance. In addition to upregulating the gene expression of heat shock proteins, such as HSP90, multiwall carbon nanotubes also contribute to heat shock protein biosynthesis [63]. In maize, CeO_2_ nanoparticles cause H_2_O_2_ to be generated excessively and HSP70 to be upregulated. By regulating stomatal opening, NPs reduce the effects of heat stress [71].

#### 4.1.1. Physiological and Biochemical Aspect

The adverse effect of cold and heat stress on plant growth and physiology are well documented. Through the application of NPs under stress conditions, plant growth and functioning can be maintained. The application of biological selenium NPs at 100 µg/mL increased plant productivity by improving plant growth, photosynthetic rate, and gas exchange at elevated temperatures [72]. In mungbean, Kareem et al. [73] reported that the application of nano-ZnO NPs at elevated temperature increased chlorophyll activity, gas exchange parameters, and enzymatic balance, which resulted in an increase in pod number, size, and total grain yield. In wheat seedlings under heat stress, the application of ZnO and TiO_2_ improved membrane stability and antioxidant defense mechanism both in root and shoot parameters [74]. The ability of nano-ZnO NPs to regulate osmatic potential and reduction in thylakoid damage by activating antioxidant defense, ensured higher plant production. ZnO NPs have also been associated with cold tolerance in rice; its application reduced oxidative stress, improved photosynthetic activity, and increased root, shoot length, and dry mass [75]. The application of NPs (nSiO_2_, nSe, nZnO, GNRs) to sugarcane, mitigated cold stress by increasing chlorophyll content which improved the photosynthetic rate and negated the impact of gas exchange parameters and oxidative activity [76]. NP application helps plants to maintain ion concentration which consequently results in membrane stability and osmotic regulation. The ability to maintain water and nutrient transport under stress conditions increases plant vegetative productivity. The prevention of enzymatic oxidative stress under stress conditions also ensures production. The production of enzymatic anti-oxidizers, i.e., SOD, POD, CAT, APX, stimulated by the application of NPs counter the oxidative stress under heat and cold stress conditions. However, the concentration of NP application is of utmost importance, because at higher levels, the toxicity caused by NPs can be very harmful [77,78]. The physiological, morphological, and genetic modifications responsible for cold and heat stress tolerance in response to nanoparticle application will also be highlighted (Table 2).

Plants are affected by heat stress in several ways, including growth, development, physiological processes, and yield. The excessive production of reactive oxygen species (ROS) in plants because of high temperatures results in oxidative stress [84]. Enzymes are required for different metabolic pathways, and their sensitivity to temperature varies. In response to heat stress, enzymes may become uncoupled from metabolic pathways, resulting in the accumulation of ROS, which primarily include singlet oxygen (^1^O_2_), superoxide radicals (O_2_), hydrogen peroxide (H_2_O_2_) and hydroxyl radicals [85]. Application of NPs help plants to improve their defense systems against high temperature (Table 2). ZnO and TiO_2_ help to improve defense by production of SOD, GPX, and reduced H_2_O_2_ content in plants. Application of nanoparticle TiO_2_ reduced H_2_O_2_ content and increased photosynthetic activity [81]. Under high temperature stress, Zn NP and Fe NP application improved the antioxidant enzyme activity in plants [86]. TiO_2_ also improved the PSII activity in plants under mild high temperature stress [87].

#### 4.1.2. Molecular Aspect

Regulation of plant stress response, as mediated by expression of genes and consequently enzymes and protein production, directly influences productivity in agricultural crops. Many genes, transcription factors and proteins are responsible for heat and cold stress tolerance. The stimulation of expression, either downregulation or upregulation, has direct consequence on plant survival. In rice, foliar application of ZnO NPs induced the chilling-induced gene expression of the antioxidative system *(OsCu/ZnSOD1, OsCu/ZnSOD2, OsCu/ZnSOD3, OsPRX11, OsPRX65, OsPRX89, OsCATA,* and *OsCATB*) and chilling-response transcription factors (*OsbZIP52, OsMYB4, OsMYB30, OsNAC5, OsWRKY76*, and *OsWRKY94*) in leaves of chilling-treated seedlings [75]. In soybean seedlings, ZnO NPs transactionally upregulated the *EREB, R2R3MYB, HSF-34, WRKY1, MAPK1, HDA3*, CAT genes which consequently increased photosynthetic pigments, proline concentration, antioxidant enzyme activity and plant yields [88]. A transcriptome study found that (50 nm) Cu-based NPs modulated genes that respond to oxidative stress, brassinosteroid biosynthesis, and root formation [89]. Cu nanoparticles of 40 nm size were studied for their ability to accumulate secondary metabolites (acetyl glucosamine, phenyl lactate, 4-aminobutyrate) that are involved in cell signaling and defense responses. Flavonoids, fatty acids, riboflavin, and amino acids were all shown to be degraded in metabolites involved in synthesis and defense responses [90]. In wheat seedlings, the application of silicon rather than Si NPs was observed to induce overexpression of *TaPIP1* and *TaNIP2* aquaporin genes at heat stress, which increased relative water content [91]. Arabidopsis thaliana seedlings grown under ZnO NPs and subjected to heat stress (37 °C) significantly enhanced heat stress-induced alleviation of *TGS-GUS* genes [92]. Nano-anatase increased Rubisco activase (RCA) mRNA concentrations and activity, resulting in improved Rubisco carboxylation and high photosynthetic carbon production rates. In maize seedlings, at root tips, the application of lanthanum oxide (La_2_O_3_) affected the expression of aquaporin genes such as *TIPs, PIPs, SIPs* and *NIPs* [93]. Different NPs are associated with up- and downregulation of many cold and heat stress inducive/regulatory transcription factors and genes. The upregulation of heat and cold stress regulating genes and transcription factors improves plant stress tolerance which enables plant physiological, molecular and biochemical modifications.

## 5. Salinity

To achieve sustainable crop production, research communities need to address soil salinity. Approximately 20% of cultivated land across the globe is under salinity threat, and this number is growing. The term saline soil refers to soil with a concentration of water-soluble salts greater than 4 dS m^−1^. With increasing urbanization and rising global food demand, farming is shifting to drier or marginal fields, leading to a reduction in land area and water input necessary to produce more food. Plants are typically affected by salinity stress through decreased soil osmotic ability, nutritional imbalances, and an increase in basic ionic toxicity (salt stress) [94]. According to Khalid et al. [95] and Ahmed et al. [96], plants’ responses to salt stress are quite complex (e.g., osmotic regulation, ion compartmentation and/or exclusion, toxic ion uptake, ROS generation, and electron transport during photosynthetic photosynthesis). It is important to note that plant responses to stress are influenced by several factors, including type, concentration, and genetic potential of the solute, as well as type and severity of the stress [97].

In addition, plant cells respond differently to salinity stress depending on the type and dose of salt. Molecular, biochemical, and physiological pathways are modulated by plants to protect themselves against salinity stress [98]. Ion haemostasis, antioxidant regulation by enzymes and non-enzymes, compatible solute accumulation and osmotic protection, hormonal regulation, changes in gene expression for stress resistance, and nitric oxide regulation, are some of these mechanisms [99]. Salt-tolerant plants, as well as the development of salt-resistant crop varieties, can help solve the problem of declining global food production by allowing farmers to breed salt-tolerant plants and develop salt-resistant crops. The conventional breeding method of inter-specific or inter-generic hybridization has had limited success in improving crop plants’ stress tolerance. In recent studies [100], various strategies have been developed to minimize the negative effects of salinity on plants. As an effective tool for improving growth and survival under salinity stress, NPs have garnered much attention in recent years [46].

A variety of developmental stages are affected by the application of NPs, both positively and negatively [101]. It has been demonstrated in different studies that NPs have beneficial effects on plants under salinity stress [101,102]. A variety of profound effects have been observed on the morphological, physiological, and biochemical properties of plant species by NPs. It has been reported that NPs can manipulate the responses of plants to salinity, as they affect hormonal concentrations, antioxidant enzyme activity, ion homeostasis, gene expression, and defense system functions [101,103]. In addition to size, shape, and concentrations of NPs applied, these effects might also vary under different environmental conditions or between different plant species [103]. Based on the concentrations and properties of the NPs [101], a variety of reports have described the toxic and negative effects of high concentrations of NPs in plants, that vary between plant tissues, growth stages, and species. As a result, NPs’ interference with various metabolic activities can be determined by their concentration, size, method of application, uptake by plants, properties, reactivity, and translocation into different tissues. Paramo et al. [104] have demonstrated toxic effects and beneficial effects on various metabolic activities.

### 5.1. NPs Mitigate Salt Stress in Plants

**Figure 3:**
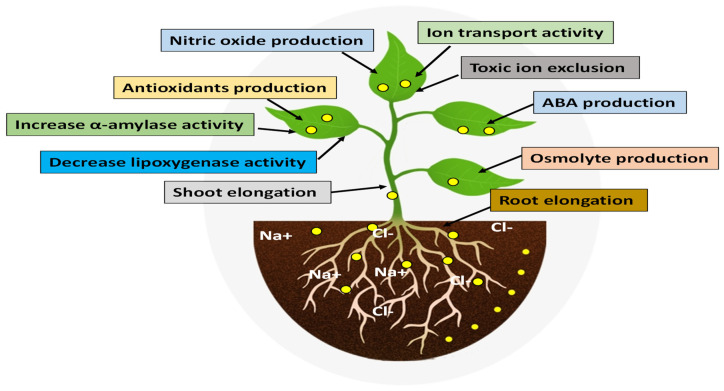
Nanomaterials can help plants mitigate the negative effect of salinity.

Plants absorb NPs through a variety of routes, primarily through roots and leaves. A second study from Khan et al. [105] suggested that NPs affect plant morphology, biochemical and physiological states, as well as molecular functions after they enter the plant. These interactions are either positive or negative, depending on the nature of the NPs and the plant species. According to Paramo et al. [104] and Tripathi et al. [106], NPs’ chemical nature, reactivity, and size could influence plant responses to NPs. Zulfiqar and Ashraf [101] demonstrated that NPs can promote plant growth and development in salt-stressed conditions. NPs enhance the salinity tolerance in plants in different ways, as shown in Figure 3. 

#### 5.1.1. Physiological and Biochemical Aspect

Photosynthesis is one of the processes most affected by salinity stress, depending on the type of plant, the salt dose, and other factors [107]. Plants can synthesize more complexes for light harvesting by foliar application of NPs, which leads to increased photosynthesis and light absorption. Several studies indicate that NPs significantly increase chlorophyll content in plants [70]. Most of the NPs available were found to increase photosynthesis by increasing the content of photosynthetic pigments in salinity-stressed plants, according to various studies that examined their effect on salinity-stressed plants [108,109]. Different parts of the cell contain manganese (such as mitochondria, chloroplasts, enzyme structures, etc.) which is also responsible for enhancing photosynthetic electron transport rates and oxygen evolution. Under abiotic stress, Mn NPs are also capable of maintaining optimal photosynthesis rates [110]. A study reported that Mn supplementation improved the membrane stability index, chlorophyll content, and nitrate reductase activity in Vigna radiata plants under salinity stress conditions [111]. Previous research has shown that the application of Cu to maize plants reduces the negative effects of salinity on water relations and photosynthesis [112].

The effects of NPs on absorption, translocation and eventual allocation of nutrients may play an imperative role in improving plant nutrition [113]. The high ratio of potassium to sodium, which is disrupted by salinity stress, has been reported to be one of the most critical factors for plant resistance to salinity stress. Plant growth under salinity stress can be improved by adding NPs to the plant and, as a result, increasing the osmotic potential within the plant [114]. According to Farhangi-Abriz and Torabian [115], nano-SiO_2_ enhanced soybean seedling growth under salt stress by increasing leaf K+ concentration. According to Perez-Labrada et al. [116], foliar application of Cu NPs enhanced tomato plant growth performance and Na+/K+ ratio after salt stress. Trachyspermum ammi plants were also found to be less sensitive to salinity stress by using Fe_2_O_3_ NPs. Using pepper plants under salinity stress, Ye et al. [110] investigated the effects of Mn NPs on the growth of pepper plants.

The production of ROS by plants in response to abiotic stresses, including salinity stress, is well known, and plants develop antioxidant enzymes to deal with excessive ROS in salinity stressed-plant cells [117]. Many studies have demonstrated that NPs increase antioxidant enzyme levels [118,119]. NPs have antioxidant properties, so they help plants overcome the conditions created by oxidative stress. In fact, Co, Fe, and Ce NPs are similar to enzyme catalase (CAT), while Ce, Mn, Cu, and Fe NPs are similar to enzyme peroxidase (POD). It was found by Wu et al. [120] that ROS-NSCC’s activity can enhance the scavenging of ROS in Arabidopsis plants treated with cerium. Pearl millet (*Pennisetum glaucum* L.) was primed with 10 mM, 20 mM, and 30 mM Ag NPs under salinity stress (0, 120, and 150 mM NaCl) by Khan et al. [105]; these NPs significantly increased growth characteristics in this plant, which was attributed to increased antioxidant enzymes such as SOD, CAT, and glutathione peroxidase (GPX), and decreased sodium to potassium ratio. Ag NPs in low concentrations have also been reported to improve antioxidant enzyme activity by Sami et al. [121]. NPs of TiO_2_ were tested on Dracocephalum moldavica under salinity stress (0, 50, 100, and 200 mM NaCl) at concentrations of 0, 50, 100 and 200 mg/L. A concentration of 100 mg/L of TiO_2_ NPs decreased the concentration of H_2_O_2_ and increased the antioxidant content [122].

#### 5.1.2. Molecular Aspect

The molecular events that occur in the plant determine its biological functions. It is imperative to evaluate potential mechanisms, and the effects on plants at the molecular level as influenced by NPs, which has been accomplished [70]. NPs cannot be effective without interfering with cellular processes and gene expression. This is because salinity stress affects gene expression which then affects plant growth by altering gene expression in various parts of the cell products. In NP-mediated root growth, *miR164* expression is decreased, which is related to auxin hormone signaling. As a result of increased *miR169* expression and decreased *miR167* expression, lateral roots can be produced, and flowering can be accelerated [123]. A foliar application of Zn NPs on rapeseed plants (*Brassica napus* L.) under salinity stress reduced the expression of some genes, such as *SKRD2, MYC* and *MPK4*, and increased the expression of other genes, such as *ARP* and *MPK* associated with physiological and hormonal responses and transcription factors, and *MYC* and *SKRD2* which are involved in abiotic stress tolerance in plant cells [124]. As a result of the application of Si NPs to *Cannabis sativa* L., the plant’s growth and molecular changes improved under salinity stress conditions [125]. In tomato plants subjected to salinity stress, proteomics analysis showed that Si affected genes involved in light-harvesting complexes, cytochrome b6f (Cytb6f) and ATP-synthesizing complexes. Siddiqui et al. [49] showed that this element was also involved in increasing the expression of *OsNAC* protein, which effectively responds to stress.

## 6. Heavy Metals

Rapid industrialization in recent decades has significantly increased the pressure on the global environment with excessive emissions of greenhouse gases. There are growing concerns about worsening global environmental conditions with an increase in droughts and water scarcity. Heavy metal pollution from the industrial sector and the continued development of urbanization threaten the ecosystem and human health [126]. Climate conditions are constantly under threat, and the challenges of a growing population ensure the difficulty in achieving food security in the 21st century.

The agriculture sector is constantly facing a challenge dealing with heavy metal deposits in soil due to rapid industrialization activities such as mining and tanneries. Agricultural practices of using excess fertilizers and pesticides have caused negative environmental and human impacts with the release of toxic chemicals and heavy metals in the air, water, and soil. Heavy metals in soil are hard to degrade, easily transferable, and highly toxic to the environment and human health, making it one of the most topical issues. Natural soil composition is adversely affected by heavy metals. The agro-biological systems of the plant are mainly damaged by chromium, cadmium, nickel, mercury, lead, and copper [127]. Heavy metals, due to their oxidative states, can be highly reactive and cause changes at the molecular and cellular level, including modifications in the physiology of the plant with the deactivation of enzymes and protein denaturation, along with replacing necessary metals and destroying membrane. These variations restrain photosynthesis and alter the enzyme activity of the plants [128]. 

Heavy metals are transported through the plasma membrane with other required nutrients with the help of metal carriers in the plant cell. Heavy metals are absorbed through the plant roots and exhibit different accumulation methods. Some plants accumulate heavy metals in their root tissues, preventing the flow to the aerial system, resulting in adequate plant growth and development. Other plants absorb heavy metals from the roots, which are moved to the shoot, and finally stored in leaves [129]. The capacity of the plant to transport the heavy metals depends on the physiological condition, vacuolar compartmentalization, and antioxidative defense system. The accumulation of metals in leaves is directly related to the atmospheric conditions. Depending on the plant mechanism, some restrict the absorption or store them in separate components to reduce the toxicity. 

Heavy metals that are harmful to plants are cadmium, chromium, copper, lead, mercury, and nickel. Each heavy metal poses a threat to plants in various ways. Water uptake imbalance is caused by high levels of cadmium, lead, and copper [130]. Chlorosis is caused by cadmium, copper, chromium, and nickel. The inhibition of metabolic activity is caused by cadmium, zinc, and chromium. Oxidative pressure and ROS generation are caused by copper, mercury, and nickel. A decrease in photosynthesis is caused by cadmium [131]. The harmful effect of these heavy metals is a common problem. The release of these is directly or indirectly due to industrial practices, which affects the food chain and reduces productivity and food quality.

### 6.1. NPs Mitigate Heavy Metals Toxicity in Plants

There is an increasing interest in the use of NPs in different industries ranging from medical treatments to the production of various products such as cosmetics and clothes. With the increase in pollution of soil, water, and air, the use of NPs in remediation with little to no harm to the environment, is gaining popularity. In comparison with bioremediation, which is more time-consuming and microbe-dependent, and chemical remediation which depends on the kinetic rate of the reaction, nanoparticle remediation is highly efficient, eco-friendly, and does not produce toxic by-products. Nanotechnology is gaining popularity in various fields due to its sustainable competitiveness and coping capabilities. The use of nanotechnology in agriculture is booming with the application of nanofertilizers and nanopesticides [132].

#### 6.1.1. Physiological and Biochemical Aspects

NPs improve chloroplast pigments and photosynthesis rate, and maintain the membrane stability in plants affected by heavy metals [133]. Hussain et al. [28] reported the application of FeO NPs for the alleviation of the effects of cadmium (Cd) in wheat. The Cd toxicity on the growth and yield were mitigated, and the morphological parameters of the wheat along with photosynthetic pigments and dry biomass of the plant were enhanced. The negative impacts of the Cd toxicity were restricted and the photosynthetic rate and growth in plants were increased. Sardar et al. [134] reported similar results in the remediation of Cd by nano-TiO_2_ in coriander with reduced Cd content, diminished oxidative injuries caused by Cd stress, and improved agronomic traits. The photosynthetic rate and growth parameters were enhanced by the application of nano-TiO_2_ in soybean [135]. An increase in biomass of summer savory because of the reduction in Cd stress was observed by the application of Si NPs by Memari-Tabrizi et al. [136]. Graphite carbon nitride was synthesized to mitigate the effects of Cd in rice [137]; a substantial increase in plant biomass and a notable reduction in Cd-induced toxicity were observed.

**Figure 4:**
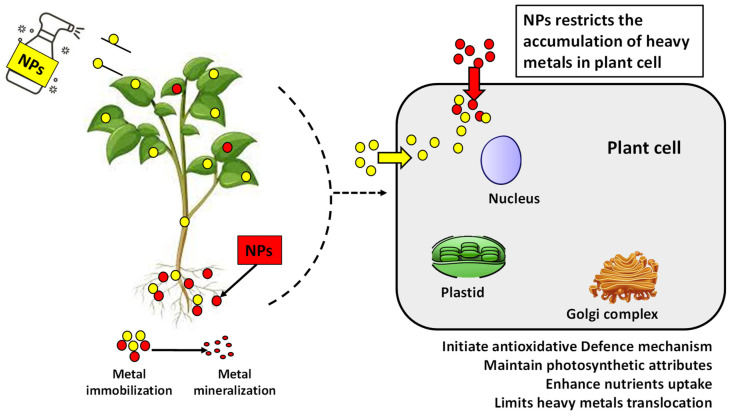
Interaction mechanism between NPs and heavy metals to mitigate heavy metal stress by reducing translocation in plants.

Different applications of NPs have been proven to remediate the oxidative stress in plants by reducing MDA and H_2_O_2_ content by regulating the antioxidant enzymes such as SOD, CAT, guaiacol, and ascorbate peroxidases [138]. NPs reduce the mobility and bioavailability of heavy metals by sticking to them, making them unavailable. NPs, due to their size, can easily move through the cell wall, and having a high surface area to volume ratio makes interacting with other molecules more accessible. The described proposed mechanism further helps to elucidate our understanding about the strategies utilized by NPs to alleviate heavy metal stress, as shown in Figure 4.

TiO_2_ NPs of different concentrations were used to reduce the toxicity of Cd in maize [139]. The toxicity was reduced with a high concentration of TiO_2_, which increased the SOD and glutathione, and upregulated metabolic pathways. Hussain et al. [140] reported the interaction of ZnO NPs on wheat for Cd alleviation. The Cd concentration was decreased, with an increase in SOD and POD activities. The alleviation of Cd uptake in soybean with nano-TiO_2_ was studied by Singh and Lee [133]. Wang et al. [71] alleviated the Cd toxicity in brassica and increased the SOD, POD, CAT, and plant biomass by the application of Cu NPs. 

Accumulation rates of arsenic (As) and Cd in rice grains were observed with the application of ZnO NPs to reduce the phytotoxicity [141]; significant decreases in As and Cd accumulation in the plant were observed. A study conducted by Bidi et al. [142] examined the application of FeO NPs on rice plants, resulting in the strengthening of the glyoxalase system and antioxidant enzymes; immobilization of As in the vacuoles and the cell walls enhanced the accumulation of the chelating agents. Fe_2_O_3_ NPs restricted the As uptake in *Vigna radiata* [143]; total antioxidant capacity was enhanced, with an increase in SOD and CAT and a decline in guaiacol peroxidase. Significant reduction in As was observed by Wang et al. [144] with the application of CuO NPs in rice, with an increase in plant biomass and antioxidant activity. 

Lead (Pb) phytoremediation is highly critical due to the Pb toxicity and complex phytoextraction. Mediation of Pb in coriander was reported by Fatemi et al. [145], with different concentrations of Si NPs. Pb stress decreased the plant biomass and vitamin C, and increased flavonoid. The adverse effects of Pb toxicity were reduced with elevated antioxidant enzyme activity. A significant increase in ryegrass biomass affected by Pb toxicity was observed by the application of nano-hydroxyapatite, by Jin et al. [146]. Chromium (Cr) phytotoxicity reduces growth in plants, with the reduction in photosynthetic pigments and chlorophyll fluorescence [147]. Increased antioxidant activity was observed with the application of Si NPs, with reduced Cr accumulation and oxidative stress, and improvement of the defense system and nutrient elements. López-Luna et al. [148] reported the use of citrate-coated magnetite NPs in wheat to study the effect on Cd and Cr. A substantial increase in root length was observed, with the accumulation of the heavy metals reduced and toxicity alleviated (Table 3).

#### 6.1.2. Molecular Aspect

The interaction of heavy metals affects the plant system mechanically and chemically, and these interactions are dependent on the plant species since each species has a specific defense mechanism to deal with stress. Cong et al. [152] reported the influence of Si NPs in reducing the uptake and toxicity of Cd in rice. Si NPs repress the genes responsible for the transportation and uptake of Cd from root to shoot which were found as low-affinity cation transporter (*LCT1*) and natural resistance-associated macrophage protein 5 (*NRAMP5*). The transport of Cd into the vacuoles gene, heavy metal ATPase 3 (*HMA3*), and silicon uptake gene, low silicon rice 1 (*LSI1*) are upregulated. The application of Si NPs increases the uptake of silicon from roots and inhibits the Cd uptake. Ahmed et al. [153] found the Cd transporter gene, such as *OsHMA2* and *OsHMA3*, responsible for heavy metal transport, and *OsLCT1* responsible for Cd translocation in the xylem and phloem. The application of FeO NPs and hydrogel NPs significantly reduced the expression of all three genes in rice. The natural resistance-associated macrophage protein (*NRAMP*) gene family is responsible for the transport of heavy metals in plant species such as rice, potato, pepper, tomato, Arabidopsis, and soybean [154]. Si NPs treatment downregulated the Cd uptake and transport genes, which improved wheat growth and alleviated the heavy metal stress [155]. Nanoscale zero-valent iron (*nZVI*) has been reported to alleviate the accumulation of heavy metals in plants and promote plant growth by downregulating genes (*IRT1, IRT2, YSL2, YSL15*) responsible for the uptake of iron and cadmium [156].

## 7. Nutrients Imbalance

Mitigating the risk of hunger and improving food security is a complex issue, with increasing challenges of rising population leading to higher food demand, contributing to food insecurity and climate change. Currently, modern agriculture feeds 6 billion people, and with our estimated population to reach up to 9.8 billion by 2050, a 70% increase in food production is required to cope with the global population. Macronutrient and micronutrient deficiencies impact the sensitivity of plants to abiotic stresses [157]. Commercial fertilizers are the most significant product used to provide extra nutrients in the soil for plant growth and development. The use of fertilizers in high concentration, due to their adequate efficiency, leads to crop damage, groundwater contamination, and soil degradation, which leads to poor product quality [158]. Moreover, fertilizers are lost due to irrigation, depending on the soil characteristics and traditional agriculture practices. An estimated 40–70% nitrogen, 80–90% phosphorus, and 50–60% potassium of the fertilizers used are lost to the surrounding environment [159]. Commercial fertilizer use is estimated to exceed 200 million tons to meet 3 billion tons of annual crop production. The reliance on commercial fertilizers is not a sustainable process to meet crop production demand [160].

Many effective approaches, such as the use of nano-fertilizers (NF), are being practiced to reduce the loss of nutrients and soil and groundwater contamination. NFs are coated with nanomaterials which control the release of nutrients depending on the plant’s requirement, and increase the nutrient use efficiency [35]. Nanotechnology is widely used in agriculture practices with nanoparticles or nanocapsules through slow-release fertilizers (SRF) or controlled-release fertilizers (CRF). In SRFs, the nutrient release is slower than normal, however, the rate of release is controlled. In contrast, CRFs are fertilizers in which the rate of release is controlled through preparation [161]. SRFs are slightly soluble in water and can be broken down by microbial activity, whereas CRFs are coated with nanomaterials which maintain the diffusion in a certain manner. High nutrient uptake by plants and reduced nutrient loss indicate a higher nutrient use efficiency [162]. 

### Physiological and Biochemical Aspect

ZnO NPs increase the germination, root length, and leaf area in *Solanum melongena* L., with a range of doses, as reported by Thunugunta et al. [163] (Table 4). Wang et al. [148] reported the application of Cu NPs in *Spinacia oleracea* L., with an increase in the fresh biomass and photosynthetic rate. Rathnayaka et al. [164] reported the application of nanonitrogen in *Oryza sativa* L., resulting in an increase in the number of tillers per plant, an increase in height, and dry biomass. The application of hydroxyapatite on *Lactuca sativa* L. resulted in an increase in phosphorus content in plants and an increase in dry biomass0 [165]. Asgari et al. [166] reported the use of nanopotassium in *Arachis hypogaea* L., resulting in an increase in shoot length, stem diameter, yield, and the number of flowers per plant. Ahmed et al. [167] reported an increase in antioxidant activity with the application of Cu NPs in *Solanum lycopersicum* L. Liu and Lal [168] reported reduced ROS activity in soybean with the application of nano-apatite.

## 8. Conclusions

It has been shown that nanomaterials currently have the potential to improve the abiotic stress tolerance of plants, as NPs display a moderately broad spectrum of actions (increasing water uptake in seeds, metabolism of starch reserves, stimulation of photosynthesis, alteration of phytohormone levels, modulation of oxidative stress or affecting nutrient absorption). However, most research has been conducted to understand one type of stressful condition. Future research needs to focus on more realistic stress conditions in real scenarios. The beneficial effects of NPs on plant health have been demonstrated by many studies; however, an exact understanding of the molecular mechanisms underlying the increased plant tolerance remain unclear. Khalid et al. [172] and Bansal et al. [173] also reported that, to enhance crop tolerance, the use of nanoparticles is one of the major strategy. Therefore, further studies are needed to determine how NPs affect the antioxidant system of plant cells, thereby improving plant tolerance to various stresses. Such an understanding may aid in the design of future smart NPs that help reduce stress and ensure sustainable agricultural production. 

The field application of many of the prepared new substances is still extremely limited due to changes in environmental conditions, soil types, plants to be treated, and most importantly, the physicochemical properties of the new metallic/nonmetallic substances. Limiting factors associated with field applications include toxicity and accumulation of NPs in crop plants. Future research on assessing the toxicological effects on model microorganisms, flora, and animals, is critical to enable field applications of nanotechnology. However, further research is needed to uncover the relevant mechanisms. Nanotechnology has also enabled plants to develop abiotic stress tolerance, but this has largely been demonstrated only at laboratory scale in the past few years. We urgently need to discuss and set up policies and regulations that are widely accepted, to facilitate the adoption of nanotechnology-enabled abiotic stress tolerance in agricultural production. Furthermore, more research needs to be conducted to investigate how nanomaterials may affect plants under abiotic stresses from the viewpoint of source-sink regulation. It would be useful to study the effects of foliar-sprayed nanomaterials on the sink capacity of plants. Overall, we believe that nanotechnology has an overly critical role to play in ensuring a sustainable agriculture community.

## Figures and Tables

**Table 1:** Impact of NPs on plants under drought stress.

NPs	Plant	Effect	Reference
ZnO	*Triticum aestivum* L.	Enhanced plant growth and mineral content in grains.	[57]
B NPs, SiO_2_ NPs and Zn NPs	*Triticum aestivum* L.	Enhanced protein contents and mitigates drought stress.	[58]
ZnO NPs	*Zea mays* L.	Enhanced yield and ameliorated antioxidative behavior.	[59]
Nano-Cu NPs	*Zea mays* L.	Upgraded the protective mechanism of maize under drought conditions.	[60]
Nano-Si NPs	*Tanacetum parthenium* L.	Improved water and phosphorus absorbing capabilities and general growth.	[61]
ZnO NPs	*Solanum lycopersicum*	Enhanced ascorbic acid and free phenols conc. along with the ameliorated activity of antioxidative enzymes.	[62]
Cu, Fe and Zn NPs	*Glycine max* (L.) Merrill	Upregulated expression of drought-sensitive genes.	[39]

**Table 2:** Impact of NPs on plants under temperature stress.

NPs	Stress	Plant	Effect	Reference
Ag NPs (50, 75 mg/L)	Heat stress (35–40 °C for 3 h/day)	*Triticum aestivum* L.	Improved plant morphological characteristics.	[79]
ZnO and TiO_2_ (1.5 and 10 ppm)	Heat stress (32 °C)	*Triticum aestivum* L.	Improved plant morphology and antioxidant defense system (SOD, GPX), reduced H_2_O_2_ content.	[74]
TiO_2_ (2, 5 ppm)	Cold stress 4 °C	*Cicer arietinum* L.	Alleviated membrane damage indexes, improved redox status.	[80]
TiO_2_	Cold stress 4 °C	*Cicer arietinum* L.	Reduced H_2_O_2_ content, increased photosynthetic activity.	[81]
Zn NPs, Fe NPs	Heat stress	*Triticum aestivum* L.	Improved yield and antioxidant enzymes activity.	[82]
ZnO NPs (10 ppm)	Heat stress	*Triticum aestivum* L.	Improved biomass, photosynthetic pigments, soluble sugars, protein and indole acetic acid (IAA) content.	[83]
ZnO NPs	Chilling stress	*Oryza Sativa* L.	Stimulated plant growth, reduced oxidative stress and gene expression of the antioxidative system.	[75]
chitosan CH NPs	Chilling stress (5 °C for 72 h)	*Musa acuminata var. Baxi*	Stimulated growth, nutrient content, photosynthesis and antioxidant enzyme activities.	[71]

**Table 3:** Impact of NPs on plants under heavy metal stress.

Plant Species	NPs	Heavy Metal	Treatment	Reference
Wheat (*Triticum aestivum*)	FeO	Cd	Decreased Cd toxicity, improved growth, yield, and chlorophyll content.	[28]
Rice (*Oryza sativa*)	ZnO	As and Cd	Decreased Cd and As concentration in roots, shoots, and leaves.	[141]
Maize (*Zea mays* L.)	TiO_2_	Cd	Decreased Cd concentration in leaves and shoots, and increased antioxidant enzyme activity.	[139]
Coriander (*Coriandrum sativum* L.)	Si	Pb	Increased plant growth and reduced Pb toxicity.	[145]
Wheat (*Triticum aestivum*)	ZnO	Cd	Decreased electrolyte leakage and increased antioxidant enzyme activity.	[140]
Pea (*Pisum sativum*)	Si	Cr	Decreased Cr phytotoxicity, accumulation, and oxidative stress markers.	[147]
Soybean (*Glycine max*)	TiO_2_	Cd	Increased photosynthetic rate and growth.	[135]
Corn (*Zea mays* L.) and broad bean seed (*Vicia faba*)	MgO	Cr, Co, Pb, Cd, and Ni	Decreased heavy metal toxicity and enhanced plant growth.	[149]
Cabbage (*brassica*)	Cu	Cd	Increased photosynthetic rate, SOD, POD, and CAT.	[71]
Rice (*Oryza sativa*)	FeO	As	Reduced As accumulation, increased Fe uptake, and restored photosynthetic pigments.	[142]
Summer savory (*Satureja hortensis* L.)	Si	Cd	Decreased Cd accumulation, and improved plant growth, total phenolic, and total flavonoid.	[136]
Mung bean (*Vigna radiata*)	Fe_2_O_3_	As	Reduced As uptake and toxicity.	[143]
Rice (*Oryza sativa*)	Cu	As	Decreased As toxicity and uptake in roots.	[150]
Wheat (*Triticum aestivum*)	Magnetite	Cd and Cr	Increased plant growth, and Cd and Cr accumulation and toxicity alleviated.	[148]
Ryegrass (*Lolium perenne* L.)	Hydroxyapatite	Pb	Increased plant biomass and Pb removal rate.	[146]
Pea (*Pisum sativum*)	Si	Cr	Reduced Cr accumulation and oxidative stress, and improved the defense system and nutrients element.	[147]
Rice (*Oryza sativa*)	Graphite carbon nitride	Cd	Elevated nitrogen content and minimized Cd-induced toxicity.	[137]
Rice (*Oryza sativa*)	Si and TiO_2_	As	Increased glutathione and phytochelatins, reduced As toxicity, and improved plant growth and tolerance.	[151]
Coriander (*Coriandrum sativum* L.)	TiO_2_	Cd	Diminished oxidative injuries and enhanced biosynthesis of proline and yield.	[134]

**Table 4:** Effect of different NFs and NPs on different crops.

Plant Species	NFs/NPs	Treatment	Reference
*Solanum melongena* L.	ZnO NPs	Increased germination, root length, and leaf area.	[163]
*Solanum lycopersicum* L.	Cu NPs	Increased antioxidant content.	[167]
*Spinacia oleracea* L.	Cu NPs	Increased fresh biomass and photosynthetic rate.	[144]
*Pisum sativum* L.	Fe NFs	Increased chlorophyll content.	[169]
*Cicer arietinum* L.	FeS2	High germination rate and crop yield.	[170]
*Oryza sativa* L.	Nano-nitrogen	Increased tiller, height, and dry biomass.	[164]
*Lactuca sativa* L.	Hydroxyapatite	Increased phosphorus content and dry biomass.	[165]
*Arachis hypogaea* L.	Nano-potassium	Increased plant growth and number of flowers per plant.	[166]
*Triticum aestivum* L.	Nano-SiO_2_	Increased water content and yield.	[171]
Soybean	Nano-apatite	Reduced ROS.	[168]

## Data Availability

Not applicable.

## References

[1] Arora N.K. (2019). Impact of climate change on agriculture production and its sustainable solutions. Environ. Sustain..

[2] Khalid M.F., Hussain S., Ahmad S., Ejaz S., Zakir I., Ali M.A., Ahmed N., Anjum M.A. (2019). Impacts of Abiotic Stresses on Growth and Development of Plants. Plant Tolerance to Environmental Stress.

[3] Jeevanandam J., Barhoum A., Chan Y.S., Dufresne A., Danquah M.K. (2018). Review on nanoparticles and nanostructured materials: History, sources, toxicity and regulations. Beilstein J. Nanotechnol..

[4] Baby A., Nazeerudeen S., Ranganath S., Samuel R.S. (2018). Toxicological impacts of nanoparticles. Emerging Trends of Nanotechnology in Environment and Sustainability.

[5] Van Wallendael A., Soltani A., Emery N.C., Peixoto M.M., Olsen J., Lowry D.B. (2019). A Molecular View of Plant Local Adaptation: Incorporating Stress-Response Networks. Annu. Rev. Plant Biol..

[6] Arif Y., Singh P., Siddiqui H., Bajguz A., Hayat S. (2020). Salinity induced physiological and biochemical changes in plants: An omic approach towards salt stress tolerance. Plant Physiol. Biochem..

[7] Heikal Y.M., El-Esawi M.A., El-Ballat E.M., Abdel-Aziz H.M.M. (2022). Applications of nanoparticles for mitigating salinity and drought stress in plants: An overview on the physiological, biochemical and molecular genetic aspects. N. Z. J. Crop Hortic. Sci..

[8] Cunningham F.J., Goh N.S., Demirer G.S., Matos J.L., Landry M.P. (2018). Nanoparticle-Mediated Delivery towards Advancing Plant Genetic Engineering. Trends Biotechnol..

[9] Feil S.B., Rodegher G., Gaiotti F., Alzate M.Y.Z., Carmona F.J., Masciocchi N., Cesco S., Pii Y. (2021). Physiological and Molecular Investigation of Urea Uptake Dynamics in *Cucumis sativus* L. Plants Fertilized with Urea-Doped Amorphous Calcium Phosphate Nanoparticles. Front. Plant Sci..

[10] Carmona F.J., Sasso G.D., Ramírez-Rodríguez G.B., Pii Y., Delgado-López J.M., Guagliardi A., Masciocchi N. (2021). Urea-functionalized amorphous calcium phosphate nanofertilizers: Optimizing the synthetic strategy towards environmental sustainability and manufacturing costs. Sci. Rep..

[11] Gaiotti F., Lucchetta M., Rodegher G., Lorenzoni D., Longo E., Boselli E., Cesco S., Belfiore N., Lovat L., Delgado-López J. (2021). Urea-Doped Calcium Phosphate Nanoparticles as Sustainable Nitrogen Nanofertilizers for Viticulture: Implications on Yield and Quality of Pinot Gris Grapevines. Agronomy.

[12] Madusanka N., Sandaruwan C., Kottegoda N., Sirisena D., Munaweera I., De Alwis A., Karunaratne V., Amaratunga G.A. (2017). Urea–hydroxyapatite-montmorillonite nanohybrid composites as slow release nitrogen compositions. Appl. Clay Sci..

[13] Marchiol L., Filippi A., Adamiano A., Degli Esposti L., Iafisco M., Mattiello A., Petrussa E., Braidot E. (2019). Influence of Hydroxyapatite Nanoparticles on Germination and Plant Metabolism of Tomato (*Solanum lycopersicum* L.): Preliminary Evidence. Agronomy.

[14] Ahmad B., Zaid A., Zulfiqar F., Bovand F., Dar T.A. (2022). Nanotechnology: A novel and sustainable approach towards heavy metal stress alleviation in plants. Nanotechnol. Environ. Eng..

[15] Pereira A.D.E.S., Oliveira H.C., Fraceto L.F., Santaella C. (2021). Nanotechnology Potential in Seed Priming for Sustainable Agriculture. Nanomaterials.

[16] Yadav S., Modi P., Dave A., Vijapura A., Patel D., Patel M., Hasanuzzaman M., Fujita M., Teixeira Filho M.C.M., Nogueira T.A.R., Galindo F.S. (2020). Effect of Abiotic Stress on Crops. Sustainable Crop Production.

[17] Salehi-Lisar S.Y., Bakhshayeshan-Agdam H. (2016). Drought stress in plants: Causes, consequences, and tolerance. Drought Stress Tolerance in Plants.

[18] Khalid M.F., Hussain S., Anjum M.A., Morillon R., Ahmad S., Ejaz S., Hussain M., Jaafar H.Z.E., Alrashood S.T., Ormenisan A.N. (2021). Physiological and biochemical responses of Kinnow mandarin grafted on diploid and tetraploid Volkamer lemon rootstocks under different water-deficit regimes. PLoS ONE.

[19] Chaudhry S., Sidhu G.P.S. (2021). Climate change regulated abiotic stress mechanisms in plants: A comprehensive review. Plant Cell Rep..

[20] Jeyasri R., Muthuramalingam P., Satish L., Pandian S., Chen J.-T., Ahmar S., Wang X., Mora-Poblete F., Ramesh M. (2021). An Overview of Abiotic Stress in Cereal Crops: Negative Impacts, Regulation, Biotechnology and Integrated Omics. Plants.

[21] Khalid M.F., Vincent C., Morillon R., Anjum M.A., Ahmad S., Hussain S. (2021). Different strategies lead to a common outcome: Different water-deficit scenarios highlight physiological and biochemical strategies of water-deficit tolerance in diploid versus tetraploid Volkamer lemon. Tree Physiol..

[22] Ortiz N., Armada E., Duque E., Roldán A., Azcón R. (2015). Contribution of arbuscular mycorrhizal fungi and/or bacteria to enhancing plant drought tolerance under natural soil conditions: Effectiveness of autochthonous or allochthonous strains. J. Plant Physiol..

[23] Gray S.B., Brady S.M. (2016). Plant developmental responses to climate change. Dev. Biol..

[24] Takahashi F., Kuromori T., Sato H., Shinozaki K. (2018). Regulatory gene networks in drought stress responses and resistance in plants. Survival Strategies in Extreme Cold and Desiccation.

[25] Ahmad J., Bashir H., Bagheri R., Baig A., Al-Huqail A., Ibrahim M.M., Qureshi M.I. (2017). Drought and salinity induced changes in ecophysiology and proteomic profile of *Parthenium hysterophorus*. PLoS ONE.

[26] Hussain S., Hussain S., Qadir T., Khaliq A., Ashraf U., Parveen A., Rafiq M. (2019). Drought stress in plants: An overview on implications, tolerance mechanisms and agronomic mitigation strategies. Plant Sci. Today.

[27] Ahmad J., Ali A.A., Baig M.A., Iqbal M., Haq I., Qureshi M.I. (2019). Role of phytochelatins in cadmium stress tolerance in plants. Cadmium Toxicity and Tolerance in Plants.

[28] Hussain A., Ali S., Rizwan M., Rehman M.Z.U., Qayyum M.F., Wang H., Rinklebe J. (2019). Responses of wheat (*Triticum aestivum*) plants grown in a Cd contaminated soil to the application of iron oxide nanoparticles. Ecotoxicol. Environ. Saf..

[29] Schulze E.-D., Beck E., Buchmann N., Clemens S., Müller-Hohenstein K., Scherer-Lorenzen M. (2019). Water Deficiency (Drought). Plant Ecology.

[30] Bielach A., Hrtyan M., Tognetti V.B. (2017). Plants under Stress: Involvement of Auxin and Cytokinin. Int. J. Mol. Sci..

[31] Zandalinas S.I., Fritschi F.B., Mittler R. (2020). Signal transduction networks during stress combination. J. Exp. Bot..

[32] Prakash V., Singh V.P., Tripathi D.K., Sharma S., Corpas F.J. (2019). Crosstalk between nitric oxide (NO) and abscisic acid (ABA) signalling molecules in higher plants. Environ. Exp. Bot..

[33] Shahid M.J., Ali S., Shabir G., Siddique M., Rizwan M., Seleiman M.F., Afzal M. (2020). Comparing the performance of four macrophytes in bacterial assisted floating treatment wetlands for the removal of trace metals (Fe, Mn, Ni, Pb, and Cr) from polluted river water. Chemosphere.

[34] Diatta A.A., Thomason W.E., Abaye O., Thompson T.L., Battaglia M.L., Vaughan L.J., Lo M., Filho J.F.D.C.L. (2020). Assessment of Nitrogen Fixation by Mungbean Genotypes in Different Soil Textures Using 15N Natural Abundance Method. J. Soil Sci. Plant Nutr..

[35] Seleiman M.F., Almutairi K.F., Alotaibi M., Shami A., Alhammad B.A., Battaglia M.L. (2020). Nano-Fertilization as an Emerging Fertilization Technique: Why Can Modern Agriculture Benefit from Its Use?. Plants.

[36] Lowry G.V., Avellan A., Gilbertson L.M. (2019). Opportunities and challenges for nanotechnology in the agritech revolution. Nat. Nanotechnol..

[37] Djanaguiraman M., Belliraj N., Bossmann S.H., Prasad P.V. (2018). High-temperature stress alleviation by selenium nanoparticle treatment in grain sorghum. ACS Omega.

[38] Ghani M.I., Saleem S., Rather S.A., Rehmani M.S., Alamri S., Rajput V.D., Kalaji H.M., Saleem N., Sial T.A., Liu M. (2022). Foliar application of zinc oxide nanoparticles: An effective strategy to mitigate drought stress in cucumber seedling by modulating antioxidant defense system and osmolytes accumulation. Chemosphere.

[39] Linh T.M., Mai N.C., Hoe P.T., Lien L.Q., Ban N.K., Hien L.T.T., Chau N.H., Van N.T. (2020). Metal-Based Nanoparticles Enhance Drought Tolerance in Soybean. J. Nanomater..

[40] Mohammadi H., Esmailpour M., Gheranpaye A. (2016). Effects of TiO_2_ nanoparticles and water deficit stress on morpho-physiological characteristics of dragonhead (*Dracocephalum moldavica* L.) plants. Acta Agric. Slov..

[41] Yang K.Y., Doxey S., McLean J.E., Britt D., Watson A., Al Qassy D., Anderson A.J. (2017). Remodeling of root morphology by CuO and ZnO nanoparticles: Effects on drought tolerance for plants colonized by a beneficial pseudomonad. Botany.

[42] Nair P.M.G., Chung I.M. (2015). Study on the correlation between copper oxide nanoparticles induced growth suppression and enhanced lignification in Indian mustard (*Brassica juncea* L.). Ecotoxicol. Environ. Saf..

[43] Liu F.Y., Xiong F.X., Fan Y.K., Li J., Wang H.Z., Xing G.M., He R. (2016). Facile and scalable fabrication engineering of fullerenol nanoparticles by improved alkaline-oxidation approach and its antioxidant potential in maize. J. Nanopart. Res..

[44] Borisev M., Borisev I., Zupunski M., Arsenov D., Pajevic S., Curcic Z., Djordjevic A. (2016). Drought impact is alleviated in sugar beets (*Beta vulgaris* L.) by foliar application of fullerenol nanoparticles. PLoS ONE.

[45] Verma S.K., Das A.K., Gantait S., Kumar V., Gurel E. (2019). Applications of carbon nanomaterials in the plant system: A perspective view on the pros and cons. Sci. Total Environ..

[46] Ahmad I., Akhtar M.S. (2019). Use of nanoparticles in alleviating salt stress. Salt Stress, Microbes, and Plant Interactions: Causes and Solution.

[47] Siddiqi K.S., Husen A. (2016). Engineered Gold Nanoparticles and Plant Adaptation Potential. Nanoscale Res. Lett..

[48] Ghasemlou F., Amiri H., Karamian R., Mirzaie-asl A. (2019). Alleviation of the effects of on drought stress *Verbascum nudicuale* by methyl jasmonate and titanium dioxide nanoparticles. Plant Physiol..

[49] Siddiqui H., Ahmed K.B.M., Sami F., Hayat S. (2020). Silicon Nanoparticles and Plants: Current Knowledge and Future Perspectives. Sustainable Agriculture Reviews.

[50] Rizwan M., Ali S., Ali B., Adrees M., Arshad M., Hussain A., ur Rehman M.Z., Waris A.A. (2019). Zinc and iron oxide nanoparticles improved the plant growth and reduced the oxidative stress and cadmium concentration in wheat. Chemosphere.

[51] Khan Z.S., Rizwan M., Hafeez M., Ali S., Adrees M., Qayyum M.F., Khalid S., Rehman M.Z.U., Sarwar M.A. (2019). Effects of silicon nanoparticles on growth and physiology of wheat in cadmium contaminated soil under different soil moisture levels. Environ. Sci. Pollut. Res..

[52] Jalil S.U., Ansari M.I. (2019). Nanoparticles and abiotic stress tolerance in plants: Synthesis, action, and signaling mechanisms. Plant Signaling Molecules.

[53] Pérez-De-Luque A. (2017). Interaction of Nanomaterials with Plants: What Do We Need for Real Applications in Agriculture?. Front. Environ. Sci..

[54] Pérez-Labrada F., Hernández-Hernández H., López-Pérez M.C., González-Morales S., BenavidesMendoza A., Juárez-Maldonado A., Tripathi D.K., Singh V.P., Chauhan D.K., Sharma S., Prasad S.M., Dubey N.K., Ramawat N. (2020). Chapter 13—Nanoparticles in plants: Morphophysiological, biochemical, and molecular responses. Plant Life under Changing Environment.

[55] Yoshida T., Fujita Y., Maruyama K., Mogami J., Todaka D., Shinozaki K., Yamaguchi-Shinozaki K.A. (2015). Four a rabidopsis AREB/ABF transcription factors function predominantly in gene expression downstream of SnRK2 kinases in abscisic acid signalling in response to osmotic stress. Plant Cell Environ..

[56] Thiruvengadam M., Gurunathan S., Chung I.-M. (2015). Physiological, metabolic, and transcriptional effects of biologically synthesized silver nanoparticles in turnip (*Brassica rapa* ssp. *rapa* L.). Protoplasma.

[57] Dimkpa C.O., Andrews J., Sanabria J., Bindraban P.S., Singh U., Elmer W.H., Gardea-Torresdey J.L., White J.C. (2020). Interactive effects of drought, organic fertilizer, and zinc oxide nanoscale and bulk particles on wheat performance and grain nutrient accumulation. Sci. Total Environ..

[58] Ahmadian K., Jalilian J., Pirzad A. (2021). Nano-fertilizers improved drought tolerance in wheat under deficit irrigation. Agric. Water Manag..

[59] Sun L., Song F., Guo J., Zhu X., Liu S., Liu F., Li X. (2020). Nano-ZnO-Induced Drought Tolerance Is Associated with Melatonin Synthesis and Metabolism in Maize. Int. J. Mol. Sci..

[60] Van Nguyen D., Nguyen H.M., Le N.T., Nguyen K.H., Nguyen H.T., Le H.M., Nguyen A.T., Dinh N.T.T., Hoang S.A., Van Ha C. (2022). Copper Nanoparticle Application Enhances Plant Growth and Grain Yield in Maize Under Drought Stress Conditions. J. Plant Growth Regul..

[61] Esmaili S., Tavallali V., Amiri B. (2020). Nano-Silicon Complexes Enhance Growth, Yield, Water Relations and Mineral Composition in *Tanacetum parthenium* under Water Deficit Stress. Silicon.

[62] El-Zohri M., Al-Wadaani N.A., Bafeel S.O. (2021). Foliar Sprayed Green Zinc Oxide Nanoparticles Mitigate Drought-Induced Oxidative Stress in Tomato. Plants.

[63] Shafqat W., Jaskani M.J., Maqbool R., Chattha W.S., Ali Z., Naqvi S.A., Haider M.S., Khan I.A., Vincent C.I. (2021). Heat shock protein and aquaporin expression enhance water conserving behavior of citrus under water deficits and high temperature conditions. Environ. Exp. Bot..

[64] Steiner J.L., Briske D.D., Brown D.P., Rottler C.M. (2018). Vulnerability of Southern Plains agriculture to climate change. Clim. Chang..

[65] Punia P., Bharti M.K., Chalia S., Dhar R., Ravelo B., Thakur P., Thakur A. (2021). Recent advances in synthesis, characterization, and applications of nanoparticles for contaminated water treatment—A review. Ceram. Int..

[66] Rizwan M., Ali S., Rehman M.Z.U., Riaz M., Adrees M., Hussain A., Zahir Z.A., Rinklebe J. (2021). Effects of nanoparticles on trace element uptake and toxicity in plants: A review. Ecotoxicol. Environ. Saf..

[67] Guha A., Vharachumu T., Khalid M.F., Keeley M., Avenson T.J., Vincent C. (2022). Short-term warming does not affect intrinsic thermotolerance but induces strong sustaining photoprotection in tropical evergreen citrus genotypes. Plant Cell Environ..

[68] Aghdam M.T.B., Mohammadi H., Ghorbanpour M. (2016). Effects of nanoparticulate anatase titanium dioxide on physiological and biochemical performance of *Linum usitatissimum* (Linaceae) under well-watered and drought stress conditions. Braz. J. Bot..

[69] Dimkpa C.O., Bindraban P.S. (2017). Nanofertilizers: New Products for the Industry?. J. Agric. Food Chem..

[70] Ali S., Mehmood A., Khan N. (2021). Uptake, Translocation, and Consequences of Nanomaterials on Plant Growth and Stress Adaptation. J. Nanomater..

[71] Wang S., Fu Y., Zheng S., Xu Y., Sun Y. (2022). Phytotoxicity and Accumulation of Copper-Based Nanoparticles in *Brassica* under Cadmium Stress. Nanomaterials.

[72] El-Saadony M.T., Saad A.M., Najjar A.A., Alzahrani S.O., Alkhatib F.M., Shafi M.E., Selem E., Desoky E.-S.M., Fouda S.E., El-Tahan A.M. (2021). The use of biological selenium nanoparticles to suppress *Triticum aestivum* L. crown and root rot diseases induced by Fusarium species and improve yield under drought and heat stress. Saudi J. Biol. Sci..

[73] Kareem H.A., Saleem M.F., Saleem S., Rather S.A., Wani S.H., Siddiqui M.H., Alamri S., Kumar R., Gaikwad N.B., Guo Z. (2022). Zinc Oxide Nanoparticles Interplay with Physiological and Biochemical Attributes in Terminal Heat Stress Alleviation in Mungbean (*Vigna radiata* L.). Front. Plant Sci..

[74] Thakur S., Asthir B., Kaur G., Kalia A., Sharma A. (2021). Zinc oxide and titanium dioxide nanoparticles influence heat stress tolerance mediated by antioxidant defense system in wheat. Cereal Res. Commun..

[75] Song Y., Jiang M., Zhang H., Li R. (2021). Zinc Oxide Nanoparticles Alleviate Chilling Stress in Rice (*Oryza sativa* L.) by Regulating Antioxidative System and Chilling Response Transcription Factors. Molecules.

[76] Elsheery N.I., Helaly M.N., El-Hoseiny H.M., Alam-Eldein S.M. (2020). Zinc Oxide and Silicone Nanoparticles to Improve the Resistance Mechanism and Annual Productivity of Salt-Stressed Mango Trees. Agronomy.

[77] Rajput V., Minkina T., Kumari A., Harish, Singh V., Verma K., Mandzhieva S., Sushkova S., Srivastava S., Keswani C. (2021). Coping with the Challenges of Abiotic Stress in Plants: New Dimensions in the Field Application of Nanoparticles. Plants.

[78] Wang P., Lombi E., Zhao F.-J., Kopittke P.M. (2016). Nanotechnology: A New Opportunity in Plant Sciences. Trends Plant Sci..

[79] Iqbal M.S., Singh A.K., Singh S.P., Ansari M.I. (2020). Nanoparticles and Plant Interaction with Respect to Stress Response. Nanomaterials and Environmental Biotechnology.

[80] Ghabel V.K., Karamian R. (2020). Effects of TiO_2_ nanoparticles and spermine on antioxidant responses of *Glycyrrhiza glabra* L. to cold stress. Acta Bot. Croat..

[81] Hasanpour H., Maali-Amir R., Zeinali H. (2015). Effect of TiO_2_ nanoparticles on metabolic limitations to photosynthesis under cold in chickpea. Russ. J. Plant Physiol..

[82] Hassan N.S., El Din T.A.S., Hendawey M.H., Borai I.H., Mahdi A.A. (2018). Magnetite and Zinc Oxide Nanoparticles Alleviated Heat Stress in Wheat Plants. Curr. Nanomater..

[83] Azmat A., Tanveer Y., Yasmin H., Hassan M.N., Shahzad A., Reddy M., Ahmad A. (2022). Coactive role of zinc oxide nanoparticles and plant growth promoting rhizobacteria for mitigation of synchronized effects of heat and drought stress in wheat plants. Chemosphere.

[84] Mittler R., Zandalinas S.I., Fichman Y., Van Breusegem F. (2022). Reactive oxygen species signalling in plant stress responses. Nat. Rev. Mol. Cell Biol..

[85] Debnath S., Chakraborty M., Kumawat R.K. (2022). Physiological Responses of Plants under High Temperature Stress. Biot. Res. Today.

[86] Tului V., Janmohammadi M., Abbasi A., Vahdati-Khajeh S., Nouraein M. (2021). Influence of iron, zinc and bimetallic Zn-Fe nanoparticles on growth and biochemical characteristics in chickpea (*Cicer arietinum*) cultivars. Poljopr. I Sumar..

[87] Dias M.C., Santos C., Pinto G., Silva A.M.S., Silva S. (2019). Titanium dioxide nanoparticles impaired both photochemical and non-photochemical phases of photosynthesis in wheat. Protoplasma.

[88] Mirakhorli T., Ardebili Z.O., Ladan-Moghadam A., Danaee E. (2021). Bulk and nanoparticles of zinc oxide exerted their beneficial effects by conferring modifications in transcription factors, histone deacetylase, carbon and nitrogen assimilation, antioxidant biomarkers, and secondary metabolism in soybean. PLoS ONE.

[89] Ali S., Rizwan M., Hussain A., Rehman M.Z.U., Ali B., Yousaf B., Wijaya L., Alyemeni M.N., Ahmad P. (2019). Silicon nanoparticles enhanced the growth and reduced the cadmium accumulation in grains of wheat (*Triticum aestivum* L.). Plant Physiol. Biochem..

[90] Zhao L., Huang Y., Hu J., Zhou H., Adeleye A.S., Keller A.A. (2016). ^1^H NMR and GC-MS Based Metabolomics Reveal Defense and Detoxification Mechanism of Cucumber Plant under Nano-Cu Stress. Environ. Sci. Technol..

[91] Younis A., Khattab H., Emam M. (2020). Impacts of silicon and silicon nanoparticles on leaf ultrastructure and TaPIP1 and TaNIP2 gene expressions in heat stressed wheat seedlings. Biol. Plant..

[92] Wu J., Wang T. (2020). Synergistic Effect of Zinc Oxide Nanoparticles and Heat Stress on the Alleviation of Transcriptional Gene Silencing in Arabidopsis thaliana. Bull. Environ. Contam. Toxicol..

[93] Yue L., Ma C., Zhan X., White J.C., Xing B. (2017). Molecular mechanisms of maize seedling response to La_2_O_3_ NP exposure: Water uptake, aquaporin gene expression and signal transduction. Environ. Sci. Nano.

[94] Ahmed M.Z., Gul B., Khan M.A., Watanabe K.N. (2016). Characterization and Function of Sodium Exchanger Genes in Aeluropus lagopoides Under NaCl Stress. Halophytes for Food Security in Dry Lands.

[95] Khalid M.F., Hussain S., Anjum M.A., Ahmad S., Ali M.A., Ejaz S., Morillon R. (2020). Better salinity tolerance in tetraploid vs diploid volkamer lemon seedlings is associated with robust antioxidant and osmotic adjustment mechanisms. J. Plant Physiol..

[96] Ahmed T., Elezz A.A., Khalid M.F. (2021). Hydropriming with Moringa Leaf Extract Mitigates Salt Stress in Wheat Seedlings. Agriculture.

[97] Khalid M.F., Morillon R., Anjum M.A., Ejaz S., Rao M.J., Ahmad S., Hussain S. (2022). Volkamer Lemon Tetraploid Rootstock Transmits the Salt Tolerance When Grafted with Diploid Kinnow Mandarin by Strong Antioxidant Defense Mechanism and Efficient Osmotic Adjustment. J. Plant Growth Regul..

[98] Khalid M.F., Hussain S., Anjum M.A., Ali M.A., Ahmad S., Ejaz S., Ali S., Usman M., Haque E.U., Morillon R. (2020). Efficient compartmentalization and translocation of toxic minerals lead tolerance in volkamer lemon tetraploids more than diploids under moderate and high salt stress. Fruits.

[99] Hanin M., Ebel C., Ngom M., Laplaze L., Masmoudi K. (2016). New Insights on Plant Salt Tolerance Mechanisms and Their Potential Use for Breeding. Front. Plant Sci..

[100] Etesami H., Glick B.R. (2020). Halotolerant plant growth–promoting bacteria: Prospects for alleviating salinity stress in plants. Environ. Exp. Bot..

[101] Zulfiqar F., Ashraf M. (2021). Nanoparticles potentially mediate salt stress tolerance in plants. Plant Physiol. Biochem..

[102] Noohpisheh Z., Amiri H., Mohammadi A., Farhadi S. (2021). Effect of the foliar application of zinc oxide nanoparticles on some biochemical and physiological parameters of *Trigonella foenum-graecum* under salinity stress. Plant Biosyst.—Int. J. Deal. All Asp. Plant Biol..

[103] Wahid I., Kumari S., Ahmad R., Hussain S., Alamri S., Siddiqui M., Khan M. (2020). Silver Nanoparticle Regulates Salt Tolerance in Wheat Through Changes in ABA Concentration, Ion Homeostasis, and DefenseSystems. Biomolecules.

[104] Paramo L.A., Feregrino-Pérez A.A., Guevara R., Mendoza S., Esquivel K. (2020). Nanoparticles in agroindustry: Applications, toxicity, challenges, and trends. Nanomaterials.

[105] Khan I., Raza M.A., Awan S.A., Shah G.A., Rizwan M., Ali B., Tariq R., Hassan M.J., Alyemeni M.N., Brestic M. (2020). Amelioration of salt induced toxicity in pearl millet by seed priming with silver nanoparticles (AgNPs): The oxidative damage, antioxidant enzymes and ions uptake are major determinants of salt tolerant capacity. Plant Physiol. Biochem..

[106] Tripathi D.K., Shweta Singh S., Singh S., Pandey R., Singh V.P., Sharma N.C., Prasad S.M., Dubey N.K., Chauhan D.K. (2017). An overview on manufactured nanoparticles in plants: Uptake, translocation, accumulation and phytotoxicity. Plant Physiol. Biochem..

[107] Hnilickova H., Kraus K., Vachova P., Hnilicka F. (2021). Salinity stress affects photosynthesis, malondialdehyde formation, and proline content in *Portulaca oleracea* L.. Plants.

[108] Alabdallah N.M., Alzahrani H.S. (2020). The potential mitigation effect of ZnO nanoparticles on [*Abelmoschus esculentus* L. *Moench*] metabolism under salt stress conditions. Saudi J. Biol. Sci..

[109] Singh P., Arif Y., Siddiqui H., Sami F., Zaidi R., Azam A., Alam P., Hayat S. (2021). Nanoparticles enhances the salinity toxicity tolerance in *Linum usitatissimum* L. by modulating the antioxidative enzymes, photosynthetic efficiency, redox status and cellular damage. Ecotoxicol. Environ. Saf..

[110] Ye Y., Cota-Ruiz K., Hernández-Viezcas J.A., Valdés C., Medina-Velo I.A., Turley R.S., Peralta-Videa J.R., Gardea-Torresdey J.L. (2020). Manganese nanoparticles control salinity-modulated molecular responses in *Capsicum annuum* L. through priming: A sustainable approach for agriculture. ACS Sustain. Chem. Eng..

[111] Shahi S., Srivastava M. (2018). Influence of foliar application of manganese on growth, pigment content, and nitrate reductase activity of *Vigna radiata* (L.) R. Wilczek under salinity. J. Plant Nutr..

[112] Iqbal M.N., Rasheed R., Ashraf M.Y., Hussain I. (2018). Exogenously applied zinc and copper mitigate salinity effect in maize (*Zea mays* L.) by improving key physiological and biochemical attributes. Environ. Sci. Pollut. Res..

[113] Kopittke P.M., Lombi E., Wang P., Schjoerring J.K., Husted S. (2019). Nanomaterials as fertilizers for improving plant mineral nutrition and environmental outcomes. Environ. Sci. Nano.

[114] Tahjib-Ul-Arif M., Sohag A.A.M., Afrin S., Bashar K.K., Afrin T., Mahamud A.G.M.S.U., Polash M.A.S., Hossain M.T., Sohel M.A.T., Brestic M. (2019). Differential Response of Sugar Beet to Long-Term Mild to Severe Salinity in a Soil–Pot Culture. Agriculture.

[115] Farhangi-Abriz S., Torabian S. (2018). Nano-silicon alters antioxidant activities of soybean seedlings under salt toxicity. Protoplasma.

[116] Pérez-Labrada F., López-Vargas E.R., Ortega-Ortiz H., Cadenas-Pliego G., Benavides-Mendoza A., Juárez-Maldonado A. (2019). Responses of tomato plants under saline stress to foliar application of copper nanoparticles. Plants.

[117] You J., Chan Z. (2015). ROS Regulation During Abiotic Stress Responses in Crop Plants. Front. Plant Sci..

[118] González-García Y., Cárdenas-Álvarez C., Cadenas-Pliego G., Benavides-Mendoza A., Cabrera-de-la-Fuente M., Sandoval-Rangel A., Valdés-Reyna J., Juárez-Maldonado A. (2021). Effect of three nanoparticles (Se, Si and Cu) on the bioactive compounds of bell pepper fruits under saline stress. Plants.

[119] Mushtaq A., Khan Z., Khan S., Rizwan S., Jabeen U., Bashir F., Ismail T., Anjum S., Masood A. (2020). Effect of Silicon on Antioxidant Enzymes of Wheat (*Triticum aestivum* L.) Grown under Salt Stress. Silicon.

[120] Wu H., Shabala L., Shabala S., Giraldo J.P. (2018). Hydroxyl radical scavenging by cerium oxide nanoparticles improves *Arabidopsis* salinity tolerance by enhancing leaf mesophyll potassium retention. Environ. Sci. Nano.

[121] Sami F., Siddiqui H., Hayat S. (2020). Impact of Silver Nanoparticles on Plant Physiology: A Critical Review. Sustainable Agriculture Reviews 41.

[122] Gohari G., Mohammadi A., Akbari A., Panahirad S., Dadpour M.R., Fotopoulos V., Kimura S. (2020). Titanium dioxide nanoparticles (TiO_2_ NPs) promote growth and ameliorate salinity stress effects on essential oil profile and biochemical attributes of *Dracocephalum moldavica*. Sci. Rep..

[123] Tolaymat T., Genaidy A., Abdelraheem W., Dionysiou D., Andersen C. (2017). The effects of metallic engineered nanoparticles upon plant systems: An analytic examination of scientific evidence. Sci. Total Environ..

[124] Hezaveh T.A., Pourakbar L., Rahmani F., Alipour H. (2019). Interactive Effects of Salinity and ZnO Nanoparticles on Physiological and Molecular Parameters of Rapeseed (*Brassica napus* L.). Commun. Soil Sci. Plant Anal..

[125] Guerriero G., Sutera F.M., Torabi-Pour N., Renaut J., Hausman J.-F., Berni R., Pennington H.C., Welsh M., Dehsorkhi A., Zancan L.R. (2021). Phyto-courier, a silicon particle-based nano-biostimulant: Evidence from cannabis sativa exposed to salinity. ACS Nano.

[126] Vila-Traver J., Aguilera E., Infante-Amate J., de Molina M.G. (2021). Climate change and industrialization as the main drivers of Spanish agriculture water stress. Sci. Total Environ..

[127] Yaashikaa P., Kumar P.S., Jeevanantham S., Saravanan R. (2022). A review on bioremediation approach for heavy metal detoxification and accumulation in plants. Environ. Pollut..

[128] Manoj S.R., Karthik C., Kadirvelu K., Arulselvi P.I., Shanmugasundaram T., Bruno B., Rajkumar M. (2020). Understanding the molecular mechanisms for the enhanced phytoremediation of heavy metals through plant growth promoting rhizobacteria: A review. J. Environ. Manag..

[129] Sassykova L., Aubakirov Y., Akhmetkaliyeva M.S., Sendilvelan S., Prabhahar M., Prakash S., Tashmukhambetova Z., Abildin T., Zhussupova A. (2020). Heavy metals accumulation in plants of the dry-steppe zone of the East Kazakhstan region. Mater. Today Proc..

[130] Thakur M., Praveen S., Divte P.R., Mitra R., Kumar M., Gupta C.K., Kalidindi U., Bansal R., Roy S., Anand A. (2022). Metal tolerance in plants: Molecular and physicochemical interface determines the “not so heavy effect” of heavy metals. Chemosphere.

[131] Jin C., Fan J., Liu R., Sun R. (2015). Single and Joint Toxicity of Sulfamonomethoxine and Cadmium on Three Agricultural Crops. Soil Sediment Contam. Int. J..

[132] Farooqi Z.H., Akram M.W., Begum R., Wu W., Irfan A. (2021). Inorganic nanoparticles for reduction of hexavalent chromium: Physicochemical aspects. J. Hazard. Mater..

[133] Ahmed T., Noman M., Ijaz M., Ali S., Rizwan M., Ijaz U., Hameed A., Ahmad U., Wang Y., Sun G. (2021). Current trends and future prospective in nanoremediation of heavy metals contaminated soils: A way forward towards sustainable agriculture. Ecotoxicol. Environ. Saf..

[134] Sardar R., Ahmed S., Yasin N.A. (2022). Titanium dioxide nanoparticles mitigate cadmium toxicity in *Coriandrum sativum* L. through modulating antioxidant system, stress markers and reducing cadmium uptake. Environ. Pollut..

[135] Singh J., Lee B.-K. (2016). Influence of nano-TiO_2_ particles on the bioaccumulation of Cd in soybean plants (Glycine max): A possible mechanism for the removal of Cd from the contaminated soil. J. Environ. Manag..

[136] Memari-Tabrizi E.F., Yousefpour-Dokhanieh A., Babashpour-Asl M. (2021). Foliar-applied silicon nanoparticles mitigate cadmium stress through physio-chemical changes to improve growth, antioxidant capacity, and essential oil profile of summer savory (*Satureja hortensis* L.). Plant Physiol. Biochem..

[137] Hao Y., Lv R., Ma C., Adeel M., Zhao Z., Rao Y., Rui Y. (2021). Graphitic carbon nitride (g-C3N4) alleviates cadmium-induced phytotoxicity to rice (*Oryza sativa* L.). Environ. Sci. Pollut. Res..

[138] Azeez N.A., Dash S.S., Gummadi S.N., Deepa V.S. (2021). Nano-remediation of toxic heavy metal contamination: Hexavalent chromium [Cr(VI)]. Chemosphere.

[139] Lian J., Zhao L., Wu J., Xiong H., Bao Y., Zeb A., Tang J., Liu W. (2020). Foliar spray of TiO_2_ nanoparticles prevails over root application in reducing Cd accumulation and mitigating Cd-induced phytotoxicity in maize (*Zea mays* L.). Chemosphere.

[140] Hussain A., Ali S., Rizwan M., Zia ur Rehman M., Javed M.R., Imran M., Chatha S.A.S., Nazir R. (2018). Zinc oxide nanoparticles alter the wheat physiological response and reduce the cadmium uptake by plants. Environ. Pollut..

[141] Ma X., Sharifan H., Dou F., Sun W. (2020). Simultaneous reduction of arsenic (As) and cadmium (Cd) accumulation in rice by zinc oxide nanoparticles. Chem. Eng. J..

[142] Bidi H., Fallah H., Niknejad Y., Tari D.B. (2021). Iron oxide nanoparticles alleviate arsenic phytotoxicity in rice by improving iron uptake, oxidative stress tolerance and diminishing arsenic accumulation. Plant Physiol. Biochem..

[143] Shabnam N., Kim M., Kim H. (2019). Iron (III) oxide nanoparticles alleviate arsenic induced stunting in Vigna radiata. Ecotoxicol. Environ. Saf..

[144] Wang Y., Lin Y., Xu Y., Yin Y., Guo H., Du W. (2019). Divergence in response of lettuce (*var. ramosa Hort*.) to copper oxide nanoparticles/microparticles as potential agricultural fertilizer. Environ. Pollut. Bioavailab..

[145] Fatemi H., Pour B.E., Rizwan M. (2020). Foliar application of silicon nanoparticles affected the growth, vitamin C, flavonoid, and antioxidant enzyme activities of coriander (*Coriandrum sativum* L.) plants grown in lead (Pb)-spiked soil. Environ. Sci. Pollut. Res..

[146] Jin Y., Liu W., Li X.-L., Shen S.-G., Liang S.-X., Liu C., Shan L. (2016). Nano-hydroxyapatite immobilized lead and enhanced plant growth of ryegrass in a contaminated soil. Ecol. Eng..

[147] Tripathi D.K., Singh V.P., Prasad S.M., Chauhan D.K., Dubey N.K. (2015). Silicon nanoparticles (SiNp) alleviate chromium (VI) phytotoxicity in *Pisum sativum* (L.) seedlings. Plant Physiol. Biochem..

[148] López-Luna J., Silva-Silva M., Martinez-Vargas S., Ricárdez O.F.M., González-Chávez M., Solís-Domínguez F., Cuevas-Díaz M. (2016). Magnetite nanoparticle (NP) uptake by wheat plants and its effect on cadmium and chromium toxicological behavior. Sci. Total Environ..

[149] Fouda A., Hassan S.E.-D., Saied E., Hamza M.F. (2021). Photocatalytic degradation of real textile and tannery effluent using biosynthesized magnesium oxide nanoparticles (MgO-NPs), heavy metal adsorption, phytotoxicity, and antimicrobial activity. J. Environ. Chem. Eng..

[150] Wang X., Sun W., Ma X. (2019). Differential impacts of copper oxide nanoparticles and Copper(II) ions on the uptake and accumulation of arsenic in rice (*Oryza sativa*). Environ. Pollut..

[151] Kiany T., Pishkar L., Sartipnia N., Iranbakhsh A., Barzin G. (2022). Effects of silicon and titanium dioxide nanoparticles on arsenic accumulation, phytochelatin metabolism, and antioxidant system by rice under arsenic toxicity. Environ. Sci. Pollut. Res..

[152] Cong W., Miao Y., Xu L., Zhang Y., Yuan C., Wang J., Zhuang T., Lin X., Jiang L., Wang N. (2019). Transgenerational memory of gene expression changes induced by heavy metal stress in rice (*Oryza sativa* L.). BMC Plant Biol..

[153] Ahmed T., Noman M., Manzoor N., Shahid M., Abdullah M., Ali L., Wang G., Hashem A., Al-Arjani A.-B.F., Alqarawi A.A. (2021). Nanoparticle-based amelioration of drought stress and cadmium toxicity in rice via triggering the stress responsive genetic mechanisms and nutrient acquisition. Ecotoxicol. Environ. Saf..

[154] Tian W., He G., Qin L., Li D., Meng L., Huang Y., He T. (2021). Genome-wide analysis of the NRAMP gene family in potato (*Solanum tuberosum*): Identification, expression analysis and response to five heavy metals stress. Ecotoxicol. Environ. Saf..

[155] Khan Z.S., Rizwan M., Hafeez M., Ali S., Javed M.R., Adrees M. (2019). The accumulation of cadmium in wheat (*Triticum aestivum*) as influenced by zinc oxide nanoparticles and soil moisture conditions. Environ. Sci. Pollut. Res..

[156] Guha T., Barman S., Mukherjee A., Kundu R. (2020). Nano-scale zero valent iron modulates Fe/Cd transporters and immobilizes soil Cd for production of Cd free rice. Chemosphere.

[157] Soares J.C., Santos C.S., Carvalho S.M.P., Pintado M.M., Vasconcelos M.W. (2019). Preserving the nutritional quality of crop plants under a changing climate: Importance and strategies. Plant Soil.

[158] El-Aziz M.A., Morsi S., Salama D.M., Elwahed M.S.A., Shaaban E., Youssef A. (2019). Preparation and characterization of chitosan/polyacrylic acid/copper nanocomposites and their impact on onion production. Int. J. Biol. Macromol..

[159] Duhan J.S., Kumar R., Kumar N., Kaur P., Nehra K., Duhan S. (2017). Nanotechnology: The new perspective in precision agriculture. Biotechnol. Rep..

[160] Usman M., Farooq M., Wakeel A., Nawaz A., Alam Cheema S.A., Rehman H.U., Ashraf I., Sanaullah M. (2020). Nanotechnology in agriculture: Current status, challenges and future opportunities. Sci. Total Environ..

[161] Salama D.M., El-Aziz M.A., Rizk F.A., Elwahed M.A. (2021). Applications of nanotechnology on vegetable crops. Chemosphere.

[162] Rahman H., Haque K.S., Khan Z.H. (2021). A review on application of controlled released fertilizers influencing the sustainable agricultural production: A Cleaner production process. Environ. Technol. Innov..

[163] Thunugunta T., Reddy A.C., Seetharamaiah S.K., Hunashikatti L.R., Chandrappa S.G., Kalathil N.C., Reddy L.R.D.C. (2018). Impact of Zinc oxide nanoparticles on eggplant (*S. melongena*): Studies on growth and the accumulation of nanoparticles. IET Nanobiotechnol..

[164] Rathnayaka R., Iqbal Y., Rifnas L., Mahendran S. (2018). Influence of Urea and Nano-Nitrogen Fertilizers on the Growth and Yield of Rice (*Oryza sativa* L.). Int. J. Res. Publ..

[165] Taşkın M.B., Şahin Ö., Taskin H., Atakol O., Inal A., Gunes A. (2018). Effect of synthetic nano-hydroxyapatite as an alternative phosphorus source on growth and phosphorus nutrition of lettuce (*Lactuca sativa* L.) plant. J. Plant Nutr..

[166] Asgari S., Moradi H., Afshari H. (2018). Evaluation of some Physiological and Morphological Characteristics of Narcissus tazatta Under BA Treatment and Nano-Potassium Fertilizer. J. Chem. Health Risks.

[167] Ahmed B., Shahid M., Khan M.S., Musarrat J. (2018). Chromosomal aberrations, cell suppression and oxidative stress generation induced by metal oxide nanoparticles in onion (*Allium cepa*) bulb. Metallomics.

[168] Liu R., Lal R. (2015). Potentials of engineered nanoparticles as fertilizers for increasing agronomic productions. Sci. Total Environ..

[169] Giorgetti L., Spanò C., Muccifora S., Bellani L., Tassi E., Bottega S., Di Gregorio S., Siracusa G., di Toppi L.S., Castiglione M.R. (2019). An integrated approach to highlight biological responses of Pisum sativum root to nano-TiO2 exposure in a biosolid-amended agricultural soil. Sci. Total Environ..

[170] Das C.K., Srivastava G., Dubey A., Roy M., Jain S., Sethy N.K., Saxena M., Harke S., Sarkar S., Misra K. (2016). Nano-iron pyrite seed dressing: A sustainable intervention to reduce fertilizer consumption in vegetable (beetroot, carrot), spice (fenugreek), fodder (alfalfa), and oilseed (mustard, sesamum) crops. Nanotechnol. Environ. Eng..

[171] Behboudi F., Sarvestani Z.T., Kassaee M.Z., Sanavi S.A.M.M., Sorooshzadeh A. (2017). Phytotoxicity of Chitosan and SiO_2_ Nanoparticles to Seed Germination of Wheat (*Triticum aestivum* L.) and Barley (*Hordeum vulgare* L.) Plants. Not. Sci. Biol..

[172] Khalid M.F., Huda S., Yong M., Lihua L., Li L., Chen Z.H., Ahmed T. (2022). Alleviation of drought and salt stress in vegetables: Crop responses and mitigation strategies. Plant Growth Regul..

[173] Bansal K., Hooda V., Verma N., Kharewal T., Tehri N., Dhull V., Gahlaut A. (2022). Stress Alleviation and Crop Improvement Using Silicon Nanoparticles in Agriculture: A Review. Silicon.

